# Reference Pose Generation for Long-term Visual Localization via Learned Features and View Synthesis

**DOI:** 10.1007/s11263-020-01399-8

**Published:** 2020-12-23

**Authors:** Zichao Zhang, Torsten Sattler, Davide Scaramuzza

**Affiliations:** 1 Robotics and Perception Group, University of Zurich, Zürich, Switzerland; 2 Czech Institute of Informatics, Robotics and Cybernetics, Czech Technical University in Prague, Prague, Czech Republic

**Keywords:** Visual localization, Benchmark construction, Learned local features

## Abstract

Visual Localization is one of the key enabling technologies for autonomous driving and augmented reality. High quality datasets with accurate 6 Degree-of-Freedom (DoF) reference poses are the foundation for benchmarking and improving existing methods. Traditionally, reference poses have been obtained via Structure-from-Motion (SfM). However, SfM itself relies on local features which are prone to fail when images were taken under different conditions, e.g., day/night changes. At the same time, manually annotating feature correspondences is not scalable and potentially inaccurate. In this work, we propose a semi-automated approach to generate reference poses based on feature matching between renderings of a 3D model and real images via learned features. Given an initial pose estimate, our approach iteratively refines the pose based on feature matches against a rendering of the model from the current pose estimate. We significantly improve the nighttime reference poses of the popular Aachen Day–Night dataset, showing that state-of-the-art visual localization methods perform better (up to 47%) than predicted by the original reference poses. We extend the dataset with new nighttime test images, provide uncertainty estimates for our new reference poses, and introduce a new evaluation criterion. We will make our reference poses and our framework publicly available upon publication.

## Introduction

Visual localization is the problem of estimating the camera pose, i.e., the position and orientation from which an image was taken, with respect to a known scene. Visual localization is a core component of many interesting applications such as self-driving cars (Heng et al. 2019) and other autonomous robots such as drones (Lim et al. 2012), as well as for augmented and virtual reality systems (Castle et al. 2008; Lynen et al. 2015).

Similar to other areas in computer vision, the availability of benchmark datasets such as Shotton et al. (2013), Valentin et al. (2016), Kendall et al. (2015), Sattler et al. (2012), Sattler et al. (2018), Badino et al. (2011) and Maddern et al. (2017) has served as a main driving force for research. Yet, there is a fundamental difference between visual localization and areas such as semantic segmentation and object detection in the way ground truth is obtained. For the latter, ground truth is provided by human annotations. However, humans are not able to directly predict highly accurate camera poses. Instead, ground truth is typically computed through a reference algorithm, e.g., Structure-from-Motion (SfM). Thus, localization benchmarks do not measure absolute pose accuracy. Rather, they measure to what degree methods are able to replicate the results of the reference algorithm. Given that the reference approach itself will produce inaccuracies and errors in the pose estimates, we use the term “reference poses” instead of “ground truth poses”.

It is crucial that the reference algorithm generates poses with a higher accuracy than the actual localization methods evaluated on a benchmark. It is thus common to provide more data to the reference algorithm compared to what is made available to the localization approaches. For example, data from other sensors such as depth (Shotton et al. 2013; Valentin et al. 2016), Lidar (Maddern et al. 2017), an external motion capture system such as Vicon (Schops et al. 2019), or additional images not available to the localization methods (Schops et al. 2019) can be used if available. This paper considers the case where only images are available. In this case, SfM is typically used as the reference algorithm, i.e., the reference poses are obtained jointly from all test images whereas localization approaches typically localize a single image at a time. This should lead to more accurate reference poses compared to what can be obtained from a single image.

In particular, we are interested in reference pose generation in the context of long-term localization, which is the problem of localizing images taken under different conditions, e.g., day–night or seasonal changes, against a scene captured under a reference condition. Given that scenes change over time, long-term localization is an important problem in practice. The main challenge in this setting is data association, i.e., establishing feature matches between images taken under different conditions. Naturally, this causes problems for generating reference poses using SfM algorithms, which themselves rely on local features such as SIFT (Lowe 2004) for data association. In previous work, we thus relied on human annotations to obtain feature matches between images taken under different conditions (Sattler et al. 2018). However, this approach is not scalable. Furthermore, human annotations of feature positions in images tend to be inaccurate, as they can easily be off by 5–10 pixels or more.

**Fig. 1:**
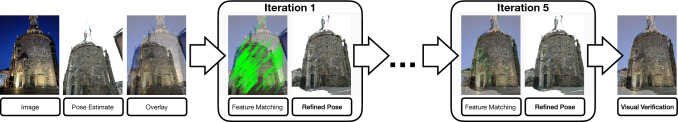
Overview of our approach: Given an image, we render a synthesized view of a 3D model from the given initial pose estimate of the image. Superimposing the rendered image over the original image provides a visual cue on the accuracy of the pose estimate. We match features extracted from the actual image and the rendering (shown as green lines connecting the corresponding positions in the overlay of the two images). This provides 2D–3D correspondences between the image and the underlying scene model. These 2D–3D matches are then used to obtain a refined estimate. Iterating this approach leads to subsequently more accurate poses (as evident from the smaller lines caused by a more accurate overlay). The final pose estimate can also be verified visually

This paper is motivated by the observation that the reference poses for the nighttime test images of the Aachen Day–Night dataset (Sattler et al. 2018, 2012), obtained from human annotations, are not accurate enough to benchmark state-of-the-art localization methods. This paper thus proposes a semi-automated approach to reference pose generation. Our method is inspired by previous work on pose verification via view synthesis (Taira et al. 2018, 2019; Torii et al. 2018) and the observation that modern learned local features (Dusmanu et al. 2019; Revaud et al. 2019) capture higher-level shape information. The latter allows feature matching between real images and 3D models, e.g., obtained via multi-view stereo (Schönberger et al. 2016). As shown in Fig. 1, our approach starts with a given initial pose estimate. It renders the 3D scene model from the current pose estimate. Feature matches between the actual and the re-rendered image are then used to refine the pose estimate. This procedure is repeated for a fixed number of iterations. Detailed experiments, for multiple ways to obtain initial poses, show that our approach yields more accurate pose estimates.

Re-rendering the image from its estimate pose enables visual inspection of the accuracy of the estimate. Using this aid, we observe that even larger differences in pose of 20cm or more can have little impact on the rendered image. This is not particularly surprising as the uncertainty of a pose estimate depends on the distance to the scene. However, it also implies that using fixed position and rotation thresholds on the pose error to measure localization accuracy (Shotton et al. 2013; Sattler et al. 2018) is not appropriate if there are significant changes in scene depth between test images. As a second contribution, we thus discuss and evaluate multiple evaluation measures that (explicitly or implicitly) use per-image uncertainty measures rather than global thresholds on pose errors.

In detail, this paper makes the following contributions: (1) we propose an approach based on view synthesis and learned features that can be used to generate reference pose for long-term visual localization benchmarks. (2) we provide a detailed experimental analysis of our approach, including studying different initialization approaches, different strategies for rendering and different features. (3) we show that the existing nighttime reference poses of the Aachen Day–Night dataset are not accurate enough to evaluate state-of-the-art long-term localization approaches. We further use our approach to obtain refined reference poses and show that current localization approaches achieve a much higher (up to 47%) pose accuracy than indicated by the original reference poses. (4) we extend the Aachen Day–Night dataset by additional nighttime test images, effectively doubling the number of available test images. We evaluate state-of-the-art localization approaches on the extended dataset and will provide a benchmark at visuallocalization.net. (5) we discuss and experimentally study additional evaluation measures. (6) we will make source code for our approach and our evaluation measures publicly available to facilitate the creation of new benchmarks. (7) we provide a concise review of current trends in the area of visual localization.

**Fig. 2:**
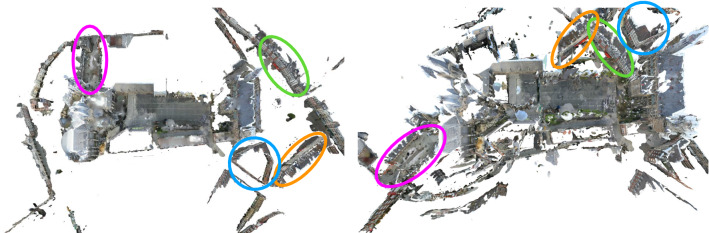
Multi-View Stereo reconstructions obtained from SfM models of the Aachen dataset using SIFT (left) and D2-Net (right) features (top-down view). D2-Net features are more robust to changes in conditions, e.g., day–night and seasonal changes, than classic SIFT features, but also produce more false positive matches. This leads to connecting unrelated scene parts during the SfM process and ultimately in an incorrect 3D model. In contrast, SIFT correctly reconstructs the scene. Some wrong placements are illustrated through colored ellipses

## Related Work

Besides discussing related work on benchmark creation for visual localization and the use of view synthesis for pose estimation and verification, this section also aims at giving an interested reader a concise overview over main trends in the area of visual localization.

Visual Localization Traditionally, most visual localization algorithms have been based on a combination of local features and a 3D scene model (Se et al. 2002; Robertson and Cipolla 2004; Li et al. 2010, 2012; Choudhary and Narayanan 2012; Irschara et al. 2009; Sattler et al. 2011; Jones and Soatto 2011; Williams et al. 2007). In most cases, the underlying 3D model is a sparse 3D point cloud constructed using SfM (Schönberger et al. 2016; Snavely et al. 2008) or SLAM (Davison et al. 2007; Mur-Artal and Tardós 2017). Each point in this model has been triangulated from two or more local image features such as SIFT (Lowe 2004) or ORB (Rublee et al. 2011). Thus, each 3D point can be associated with one or more local image descriptors. 2D–3D correspondences between local features in a query image and 3D model points can be found using nearest neighbor search in the descriptor space. In turn, these 2D–3D matches can be used to estimate the camera pose of the query image by applying an *n*-point pose solver (Haralick et al. 1994; Kukelova et al. 2013, 2010; Larsson et al. 2017; Albl et al. 2016; Kneip et al. 2011; Fischler and Bolles 1981) inside a hypothesize-and-verify framework such as RANSAC (Fischler and Bolles 1981) and its variants (Chum and Matas 2008; Lebeda et al. 2012; Raguram et al. 2013). Research on such 3D *structure-based* methods has mostly focused on scalability, e.g., by accelerating the 2D–3D matching stage (Li et al. 2010, 2012; Choudhary and Narayanan 2012; Sattler et al. 2017; Donoser and Schmalstieg 2014; Lim et al. 2012; Jones and Soatto 2011; Cheng et al. 2019) and the use of image retrieval (Irschara et al. 2009; Sattler et al. 2012; Sarlin et al. 2019; Taira et al. 2018; Liu et al. 2017; Cao and Snavely 2013), by reducing memory requirements through model compression (Li et al. 2010; Cao and Snavely 2014; Camposeco et al. 2019; Lynen et al. 2015; Dymczyk et al. 2015), or by making the pose estimation stage more robust to the ambiguities encountered at scale (Li et al. 2012; Zeisl et al. 2015; Svärm et al. 2017; Toft and Larsson 2016; Alcantarilla et al. 2011; Aiger et al. 2019).

Such approaches are computationally too complex for mobile devices with limited resources, e.g., robots and smart phones. In order to achieve real-time localization on such devices, non-real-time global localization against a pre-built map is combined with real-time local camera pose tracking (Mur-Artal and Tardós 2017; Middelberg et al. 2014; Lynen et al. 2015; Schneider et al. 2018; Kasyanov et al. 2017; DuToit et al. 2017; Jones and Soatto 2011; Ventura et al. 2014). To this end, results from the localization process (most often 2D–3D inliers) are integrated into visual(-inertial) odometry or SLAM to prevent drift in the local pose estimates.

Structure-based approaches rely on underlying 3D models, which are expensive to build at scale and costly to maintain (Sattler et al. 2017). Alternatively, the absolute pose of a query image can be estimated from the relative poses to database images with known poses (Zhang and Kosecka 2006; Zhou 2019) or 2D–2D matches with multiple database images (Zheng and Changchang 2015). It can also be estimated using local SfM models computed on the fly (Sattler et al. 2017).

Instead of explicitly using an underlying 3D model, *absolute pose regression* train a CNN to directly regress the camera pose from an input image (Brahmbhatt et al. 2018; Clark et al. 2017; Huang et al. 2019; Kendall et al. 2015; Kendall and Cipolla 2017; Melekhov et al. 2017; Naseer 2017; Radwan et al. 2018; Valada et al. 2018; Walch et al. 2017; Xue et al. 2019). However, they are not consistently more accurate than simple image retrieval baselines (Arandjelović et al. 2016; Torii et al. 2018, 2011) that approximate the pose of a query image by the poses of the top-retrieved database images (Sattler 2019). Furthermore, these approaches need to be trained specifically per scene. The latter problem can be overcome by *relative pose regression* techniques (Balntas et al. 2018; Ding et al. 2019; Laskar et al. 2017; Zhou 2019; Saha and Varma 2018), which train CNNs to predict relative poses. In combination with image retrieval against a database of images with known poses, these relative poses can be used for visual localization. While recent work shows promising results (Ding et al. 2019; Saha and Varma 2018; Zhou 2019), relative pose regression techniques do not yet achieve the same level of pose accuracy as methods explicitly based on 2D–3D matches.

Rather than learning the full localization pipeline, *scene coordinate regression* algorithms only replace the 2D–3D matching stage through a machine learning algorithm, typically either a random forest (Shotton et al. 2013; Cavallari et al. 2017, 2019a, b; Meng et al. 2017, 2018; Massiceti et al. 2017; Valentin et al. 2015, 2016) or a CNN (Brachmann et al. 2017; Brachmann and Rother 2018, 2019, 2020; Massiceti et al. 2017; Yang et al. 2019; Zhou et al. 2020). For a given patch from an image, these methods predict the corresponding 3D point in the scene. The resulting set of 2D–3D matches can then be used for camera pose estimation. Scene coordinate regression techniques constitute the state-of-the-art in terms of pose accuracy in small scenes. However, they currently do not scale well to larger scenes. For example, ESAC (Brachmann and Rother 2019), a state-of-the-art scene coordinate regression technique, localizes 42.6% of all daytime query images of the Aachen Day–Night dataset (Sattler et al. 2012, 2018) within errors of 25 cm and 5$^\circ$. In contrast, SIFT-based Active Search (Sattler et al. 2017), a classical structure-based method, localizes 85.3% within the same error thresholds.

Learned Local Features State-of-the-art approaches for long-term localization (Dusmanu et al. 2019; Sarlin et al. 2019; Germain et al. 2019; Larsson et al. 2019; Stenborg et al. 2018; Yang et al. 2020; Benbihi et al. 2019; Taira et al. 2018, 2019) are based on local features and explicit 3D scene models.^1^ Classical handcrafted features such as ORB (Rublee et al. 2011), SIFT (Lowe 2004), and SURF (Bay et al. 2008) struggle to match features between images taken under strongly differing viewing conditions, e.g., day and night or seasonal changes. Thus, long-term localization approaches typically use machine learning, both for image retrieval (Arandjelović et al. 2016; Noh et al. 2017; Radenović et al. 2019) and for local features (Ono et al. 2018; DeTone et al. 2018; Benbihi et al. 2019; Noh et al. 2017; Yang et al. 2020; Dusmanu et al. 2019).

Traditionally, local feature learning has focused on learning feature descriptors (Balntas et al. 2016; Brown et al. 2011; Ebel et al. 2019; Mishchuk et al. 2017; Simonyan et al. 2014; Simo-Serra et al. 2015; Tian et al. 2017, 2019). However, it has been shown that the local feature detector often is the limiting factor (Taira et al. 2018; Torii et al. 2018; Sattler et al. 2018; Germain et al. 2019). Thus, recent work trains feature detectors and descriptors jointly (Benbihi et al. 2019; DeTone et al. 2018; Ono et al. 2018; Yang et al. 2020; Wang et al. 2020; Noh et al. 2017), leading to state-of-the-art feature matching performance for images taken under strongly differing conditions. Interestingly, using deeper layers of neural networks pre-trained on ImageNet (Deng et al. 2009) to define both feature detector and descriptor leads to very competitive performance (Benbihi et al. 2019; Dusmanu et al. 2019). Equally important, such features are very robust to changes in different conditions, even though this might come at a price of more false positives (cf. Fig. 2). We use this robustness to establish correspondences between real images and renderings of 3D models and the resulting 2D–3D matches to compute reference poses for benchmarking long-term visual localization. In addition, we benchmark state-of-the-art long-term localization approaches (Sarlin et al. 2019; Germain et al. 2019; Revaud et al. 2019; Dusmanu et al. 2019) based on local features using our reference poses.

Semantic Visual Localization Besides using learned features that are more robust to changes in viewing conditions, long-term localization approaches also use semantic image segmentation (Budvytis et al. 2019; Garg et al. 2019; Larsson et al. 2019; Stenborg et al. 2018; Schönberger et al. 2018; Seymour et al. 2019; Shi et al. 2019; Taira et al. 2019; Toft et al. 2017, 2018; Wang et al. 2019; Yu et al. 2018). These methods are based on the observation that the semantic meaning of scene elements, in contrast to their appearance, is invariant to changes. Semantic image segmentations are thus used as an invariant representation for image retrieval (Arandjelović and Zisserman 2014; Toft et al. 2017; Yu et al. 2018), to verify 2D–3D matches (Budvytis et al. 2019; Larsson et al. 2019; Stenborg et al. 2018; Toft et al. 2018) and camera pose estimates (Shi et al. 2019; Stenborg et al. 2018; Taira et al. 2019; Toft et al. 2018), for learning local features (Garg et al. 2019; Schönberger et al. 2018), and as an additional input to learning-based localization approaches (Budvytis et al. 2019; Seymour et al. 2019; Wang et al. 2019).

View Synthesis As shown in Fig. 1, our approach iteratively renders a 3D model from a camera pose estimate and uses matches between the rendering and the actual image to refine the pose. Our approach takes inspiration from previous work on using view synthesis for pose estimation and verification. (Sibbing et al. 2013; Shan et al. 2014) render detailed laser scans (Sibbing et al. 2013) respectively dense Multi-View Stereo point clouds (Shan et al. 2014) from new perspectives. They show that SIFT feature matching between the renderings and actual images is possible if both were taken from very similar poses. Torii et al. (2018) shows that view synthesis from very similar viewpoints (obtained from depth maps) improves SIFT feature matching between day and night images. Aubry et al. (2014) learns features that can be matched between paintings and renderings of a 3D model. Valentin et al. (2016) first learns a randomized decision forest (RDF) and a hierarchical navigation graph using synthesized images (rendered from reconstructed scene models) and then uses the RDF and the graph for efficient and gradient-free localization of new query images. In these works, view synthesis is used to create novel viewpoints in a given scene in order to enable camera pose estimation at all. In contrast, this paper focuses on using view synthesis to refine an initial pose estimate and to use it for generating reference poses for a long-term localization benchmark. Thus, the contributions of this paper center around a detailed experimental evaluation of the use of view synthesis to improve pose accuracy rather than on proposing a new method.

Taira et al. (2018, 2019) use view synthesis for automated pose verification. To this end, they render a dense laser scan point cloud from a set of given poses. They densely extract descriptors from each rendering and compare each descriptor against a descriptor extracted at the same pixel in the original image to compute an image-level similarity score. This score is then used to select the pose that best explains the input image. In contrast, this paper uses view synthesis to refine the camera pose estimates. While Taira et al. (2018, 2019) automate pose estimation, their approach still has room for improvement, even if additional information such as semantics is used (Taira et al. 2019). Thus, we use the rendering for visual inspection of the poses rather than automating the verification process.

Armagan et al. (2017) utilizes synthesized views for improving an initial pose estimate with respect to a 2.5D street map (containing only the outline of the buildings). Given a rendered view of the 2.5D map and a semantic segmentation of the image, they combine two networks and a line search strategy to compute a 3DoF pose correction (horizontal translation and yaw) to the initial pose. Then the improved pose is used as the new input, and the correction procedure is applied iteratively. This paper uses a similar strategy of iterative synthesis and correction but differs in several aspects. Armagan et al. (2017) focuses on the geolocalization in urban environment, while we aim at providing a general tool for creating accurate visual localization benchmarks. This also results in the different choices of the input modality: we choose to use SfM models instead of 2.5D maps, which are specific to urban environments. Moreover, our method is more generic in that it is able to correct the poses in 6DoF instead of 3DoF.

Visual Localization Benchmarks This paper considers the visual localization problem, i.e., the task of computing the full camera pose for a given image. Closely related is the visual place recognition problem of determining which place is visible in a given image, without necessarily estimating its camera pose. However, we will not discuss pure place recognition datasets that do not provide full 6DoF camera poses such as Chen et al. (2017); Sünderhauf et al. (2015), Torii et al. (2018, 2015) and Milford and Wyeth (2012).

Early localization benchmarks used SfM to reconstruct scenes from internet photo community collections such as Flickr. Query images were then obtained by removing some images from the reconstruction, together with all 3D points visible in only one of the remaining images (Li et al. 2010). Examples for this approach to benchmark creation are the Dubrovnik, depicting the old city of Dubrovnik (Croatia), Rome (Li et al. 2010) and Landmarks 1k (Li et al. 2012) datasets. The latter two datasets consists of individual landmarks in Rome respectively around the world. The same approach was later also used for images taken under more controlled conditions, e.g., the crowd-sourced Arts Quad (Crandall et al. 2011; Li et al. 2012) dataset, the scenes from the Cambridge Landmarks (Kendall et al. 2015) benchmark, and the San Francisco SF-0 (Chen et al. 2011; Li et al. 2012; Sattler et al. 2017) dataset. Similarly, RGB-D SLAM algorithms (Newcombe et al. 2011; Dai et al. 2017) were used to obtain reference poses for the 7Scenes (Shotton et al. 2013) and 12Scenes (Valentin et al. 2016) datasets. Both depict small indoor scenes captured with RGB-D sensors.

Long-term localization benchmarks (Sattler et al. 2018; Carlevaris-Bianco et al. 2016; Balntas et al. 2019) typically use images captured under a reference condition to represent the scene while images taken under different conditions are used as query. SLAM and SfM algorithms depend on data association between images. Thus, they tend to fail if images were taken under too dissimilar conditions. Using image sequences and/or multi-camera systems can allow using SLAM and SfM algorithms under stronger viewing condition changes. The former exploits the fact that it is not necessary to find matches between each query image and a reference image. Rather, finding enough matches for some query images is sufficient to register an entire sequence. The latter exploit the fact that a larger field-of-view typically leads to more matches. Both the SILDa (Balntas et al. 2019) and (extended) CMU Seasons (Badino et al. 2011; Sattler et al. 2018) use sequences and multi-camera systems. SILDa depicts a single building block in London, UK under diferent conditions. The (extended) CMU Seasons dataset was constructed from images collected in and around Pittsburgh, US over the span of a year. For the (extended) CMU Seasons, additional humanly annotated matches were used in areas where cross-seasonal matching failed (Sattler et al. 2018). Human annotations were also used for the Mall (Sun et al. 2017) dataset to obtain initial pose estimates of test images with respect to a laser scan.

Manually annotated matches are often not very precise (Sattler et al. 2017). If available, additional sensors such as Lidar can be used to avoid the need for human annotations. The RobotCar Seasons (Maddern et al. 2017; Sattler et al. 2018), depicting the city of Oxford, UK under various seasonal conditions, and the University of Michigan North Campus Long-Term Vision and LIDAR (Carlevaris-Bianco et al. 2016) datasets use Lidar data to obtain reference poses. However, human intervention might still be necessary if the scene geometry changes (Sattler et al. 2018).

The Aachen Day–Night (Sattler et al. 2012, 2018) depicts the old inner city of Aachen, Germany. The 3D model of the scene was reconstructed from daytime images using SfM. Similarly, reference poses for daytime query images were also obtained using SfM. Since additional sensor data is not available and since SfM failed to provide reference poses (Sattler et al. 2018), manual annotations were used for a set of nighttime query images. To this end, a daytime image taken from a similar viewpoint was selected for each nighttime query. The pixel positions corresponding to 10–30 3D points visible in the daytime image were then annotated manually. Sattler et al. (2018) estimated that the median mean position accuracy for the nighttime images is between 30 and 40 cm. However, in this paper, we show that the pose estimates are actually often worse. This observation motivates our approach for refining the original reference poses. We show that the refined poses are more accurate and are thus more suitable to measure the performance of state-of-the-art localization techniques. While this paper focuses on the Aachen Day–Night dataset, our approach is not specific to it and can be applied on other datasets as well. As described in Sect. 5.1, it uses the same information as required for building SfM models, which is available in many visual localization benchmarks. The scene models used in our approach, namely a SfM model and a dense mesh, can be generated using publicly available software packages (e.g., COLMAP in our setup).

## Reference Pose Generation

Typically, a visual localization dataset provides a set of images ${\mathcal {I}}: \{I_i\}_{i=1}^{N}$ and the corresponding reference poses ${\mathcal {T}}: \{\mathtt {{T}}_i\}_{i=1}^{N}$ in a 3D model ${\mathcal {M}}$. Our goal is to know whether the poses ${\mathcal {T}}$ are accurate (verification) and get more accurate reference poses if necessary (refinement). Since each image in a visual localization dataset is usually treated individually, we consider a single image $I$ and its (potentially inaccurate) pose $\mathtt {{T}}$ in this section. $\mathtt {{T}}$ represents the camera pose with respect to the model ${\mathcal {M}}$. More specifically, $\mathtt {{T}}$ is a $4\times 4$ transformation matrix:

1 $$\begin{aligned} \mathtt {{T}}= \begin{bmatrix} \mathtt {R}&{} \mathbf {{c}}\\ \mathtt {0}_{1\times 3} &{} 1 \end{bmatrix}, \end{aligned}$$

and $\mathbf {{p}}= \mathtt {R}\cdot {}_{c}\mathbf {{p}}+ \mathbf {{c}}$ converts point coordinates in the camera frame ${}_{c}\mathbf {{p}}$ to the coordinates in the model.

Given the 3D model ${\mathcal {M}}$, we first render a synthesized view $I^{r}$ (or multiple rendered images) at pose $\mathtt {{T}}$ (Sect. 3.1). Then learned features are extracted and matched between the actual image $I$ and the synthesized image $I^r$. By analyzing the matched features, denoted as $\{\mathbf {u}_l\}_{l=1}^{N_f}$ and $\{\mathbf {u}_l^r\}_{l=1}^{N_f}$ for the actual and rendered images respectively, we can determine whether the pose $\mathtt {{T}}$ is accurate (Sect. 3.2). Finally, we can back-project the 2D features from the rendered view $\{\mathbf {u}_l^r\}_{l=1}^{N_f}$ to the 3D model ${\mathcal {M}}$ to get a set of 3D points $\{\mathbf {{p}}_l^r\}_{l=1}^{N_f}$. From the 2D–3D correspondences $\{\mathbf {u}_l\}_{l=1}^{N_f}$ and $\{\mathbf {{p}}_l^r\}_{l=1}^{N_f}$, we can calculate a more accurate pose $\mathtt {{T}}^{r}$ for the actual image (Sect. 3.3). The aforementioned process is repeated several times to get more accurate poses (cf. Fig. 1). We also discuss different methods to quantify the uncertainties of the resulting poses (Sect. 3.4), which are useful for defining localization accuracy metrics (cf. Sect. 4.1).

For simplicity of presentation, we assume that all the 2D features $\{\mathbf {u}_l^r\}_{l=1}^{N_f}$ have a valid back-projection in ${\mathcal {M}}$ and all the 3D points $\{\mathbf {{p}}_l^r\}_{l=1}^{N_f}$ are inliers in the refinement process. In practice, we remove 2D features with invalid depth (e.g., due to an incomplete the model ${\mathcal {M}}$) and reject outliers using LO-RANSAC (Lebeda et al. 2012). For simplicity, we assume that the features are ordered based on matches: for a feature $\mathbf {u}_l$ in the real image, the corresponding matching feature in a rendering is $\mathbf {u}_l^r$.

### Rendering Synthesized Views

There are different methods to render synthesized views from a pose $\mathtt {{T}}$ with respect to a scene model ${\mathcal {M}}$. In this work, we investigate view synthesis from two different scene models: a 3D point cloud with SIFT descriptors and a 3D mesh. In the process of generating reference poses using SfM, the scene is typically reconstructed as a 3D point cloud, where each point is associated with a descriptor, e.g., SIFT. A 3D mesh can be further generated using Multi-View Stereo. Therefore, these two models are readily available from the standard process for generating reference poses.

To render images from a 3D mesh, there are various off-the-shelf renderers that can be used. As for a point cloud with descriptors, we follow Pittaluga et al. (2019) and train a CNN to reconstruct the images from such a scene representation. The network uses a U-Net architecture (Ronneberger et al. 2015). The input to the network is a 3D tensor of size $h \times w \times 129$, where *h* and *w* are the height and width of the image to be synthesized. The 129 channels consists of a depth channel and one channel per byte in the SIFT descriptor (128 bytes). The input is constructed by finding and projecting the visible points in the point cloud to the pose to render and then filling the input tensor at the pixel coordinates where there is a projected 3D point. The output of the network is the synthesized image at a given pose. For details of the method (e.g., training and evaluation), we refer the reader to Pittaluga et al. (2019). While each rendering technique alone is sufficient in certain cases, combining the two rendering methods utilizes the information from different scene models and results in the best performance in our experiment (cf. Sect. 5.4).

### Matching Features with Synthesized Views

To extract and match features between the real images $I$ and the rendered images $I^{r}$, we choose to use learning-based local features. This is due to the fact that the rendered images usually have large appearance change compared with the real night images. Traditional features, such as SIFT, rely on low level image statistics and are not robust to day–night condition change and rendering artifacts. In particular, we choose to use the D2-Net feature (Dusmanu et al. 2019) in our pipeline, which uses a single CNN for joint feature detection and description and achieves state-of-the-art matching performance in challenging conditions.

**Fig. 3:**
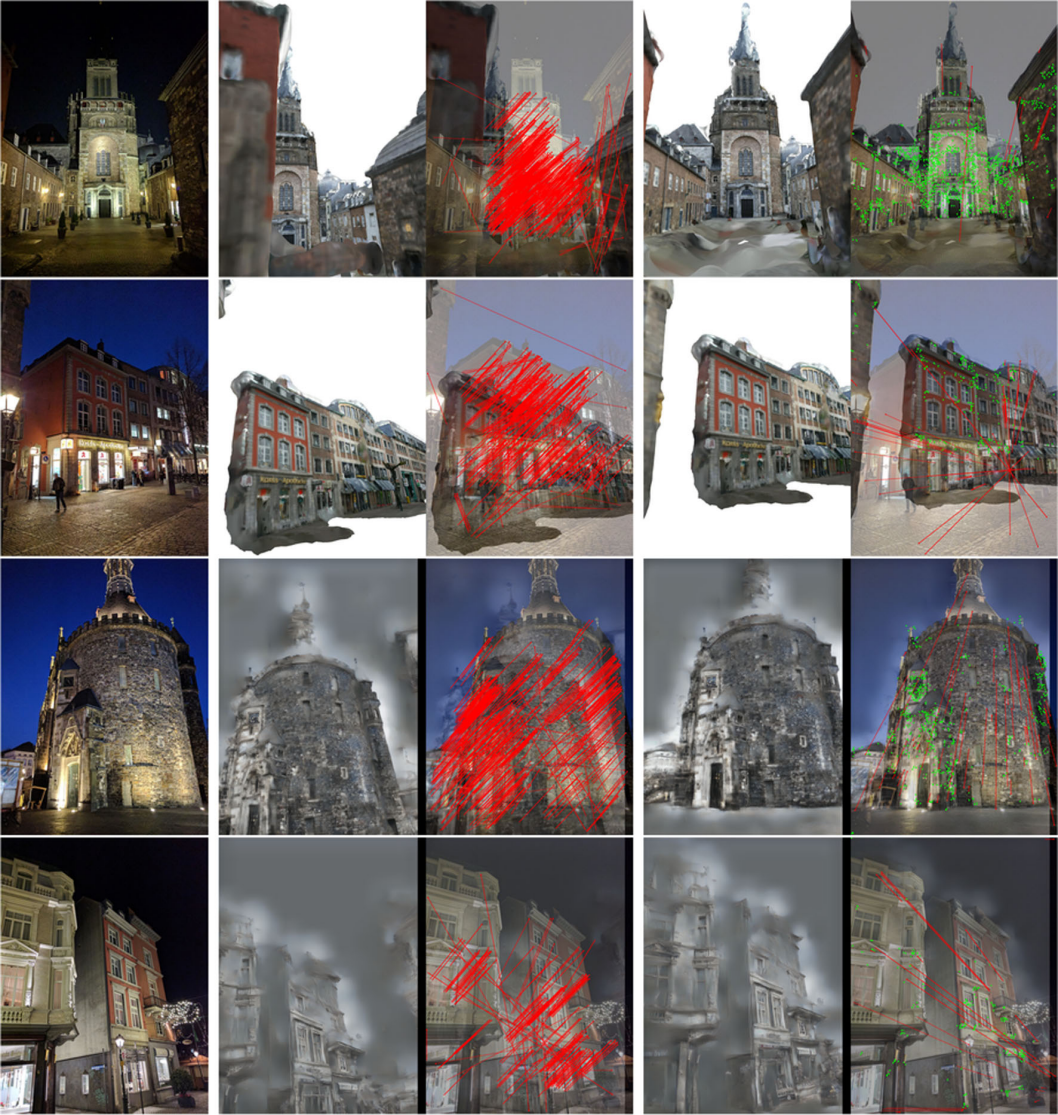
Comparison of images rendered from the original and refined (ours) reference poses of the nighttime images in Aachen Day–Night dataset. First column: nighttime images; Second column: images rendered from the existing reference poses, overlay of the rendering and the image together with D2-Net matches between the two; Third column: images rendered from our refined poses and the corresponding overlays with D2-Net matches. The top two rows render a Multi-View Stereo (MVS) mesh and the bottom two use Structure-from-Motion inversion (Pittaluga et al. 2019) (invSfM). The colored lines visualize D2-Net feature matches. Green is used to indicate that the 2D location difference between a feature in the real image and its match in the rendered image is below 20 pixel

For the images rendered using the two rendering techniques, we extract and match features between each rendered image and the real image individually. We then directly aggregate the feature matches obtained from both rendered images for the next step. Note that after obtaining the 2D feature matches, we can already verify whether there exists pose errors in the reference poses by checking the matching locations in the rendered and real images (cf. Figs. 3 and 8 for large and small pose errors respectively): if the real and rendered images are taken from the same pose, the two features $\mathbf {u}_l$ and $\mathbf {u}_l^r$ should be found at identical 2D positions (up to noise in the feature detection stage). Similarly, a large 2D distance $||\mathbf {u}_l - \mathbf {u}_l^r||_2$ is indicative for a significant difference in pose.

### Refining Reference Poses

Given $N_f$ matched features $\{\mathbf {u}_l\}_{l=1}^{N_f}$ and $\{\mathbf {u}_l^r\}_{l=1}^{N_f}$ between the real and rendered images, we first back-project the features in the rendered images to ${\mathcal {M}}$ to get the corresponding 3D points as $\{\mathbf {{p}}_l^r\}_{l=1}^{N_f}$

2 $$\begin{aligned} \mathbf {{p}}^r_l = \pi ^{-1}(\mathbf {u}_l^r, \mathtt {{T}}, K, D, {\mathcal {M}}), \end{aligned}$$

where $\pi :\mathbf {{p}}\rightarrow \mathbf {u}$ is the camera projection function and $\pi ^{-1}$ the inverse. *K* and *D* are the intrinsics and distortion parameters respectively. In practice, we get the depth map at $\mathtt {{T}}$ in the process of rendering images from the 3D mesh, and the depth at $\mathbf {u}_l$ can be directly read from the depth map. After finding the 3D points, the refined reference pose $\mathtt {{T}}^r$ can be computed by solving a nonlinear least-squares problem

3 $$\begin{aligned} \mathtt {{T}}^r = \underset{\mathtt {{T}}}{{{\,\mathrm{arg\,min}\,}}} \sum _{l=1}^{N_f} \Vert \pi (\mathbf {{p}}^r_l, \mathtt {{T}}, K, D) - \mathbf {u}_l \Vert ^2. \end{aligned}$$

We minimize (3) over the inliers of a pose obtained by LO-RANSAC. Note that it is possible to additionally refine *K* and *D* in the above optimization. This could help correct errors in the intrinsics and distortion parameters but potentially make the optimization problem less stable (e.g., when there are few feature matches). In addition, relatively accurate *K* and *D* values are required for rendering reasonable synthesized views for successful feature matching, regardless of whether they are refined in the optimization.

### Uncertainty Quantification

To use the refined pose $\mathtt {{T}}^r$ for evaluating localization accuracy, it is also important to quantify the uncertainty of the refined pose for meaningful interpretation of the difference between the pose to evaluate and the reference pose. For example, if two poses to be evaluated are both within the uncertainty range of the reference pose, they should be considered equally accurate, regardless of their absolute differences with respect to the reference pose. It is thus a typical practice to consider the uncertainty of the reference poses in the accuracy evaluation of visual localization methods (cf. Sect. 4.1). Next, we introduce two commonly used methods for covariance estimation (Hartley and Zisserman 2003, Ch. 5) and a sampling strategy explored in this paper.

First Order Approximation For the nonlinear least squares problem (3), the covariance of $\mathtt {{T}}^r$ can be computed by $\varvec{\Sigma }_{\mathtt {{T}}^r} = (\sum _{l=1}^{N_f} \mathtt {{J}}^\top _{l} \varvec{\Sigma }^{-1}_{\mathbf {u}} \mathtt {{J}}_{l})^{-1},$ where $\mathtt {{J}}_{l} = \partial \mathbf {u}_l / \partial \mathtt {{T}}$ is the Jacobian (evaluated at $\mathtt {{T}}^r$) of the *l*th landmark observation with respect to the camera pose,^2^ and $\varvec{\Sigma }_{\mathbf {u}}$ is the covariance of the landmark observation $\mathbf {u}$. $\varvec{\Sigma }_{\mathbf {u}}$ is usually assumed to be a diagonal matrix and the same for each observation. Then the uncertainty of the camera position and rotation, denoted as $\delta ^c$ and $\delta ^r$, can be calculated from positions and rotations sampled from a multivariate Gaussian distribution ${\mathcal {N}}({\mathbf {0}}, \varvec{\Sigma }_{\mathtt {{T}}^r})$:

4 $$\begin{aligned} \delta ^c = \text {median}\left( \{\vert \delta {\mathbf {c}}_{n}\vert \}_{n=1}^{N_s}\right) \quad \delta ^r = \text {median}\left( \{\vert \delta {\mathbf {r}}_{n}\vert \}_{n=1}^{N_s}\right) , \end{aligned}$$

where $\vert \cdot \vert$ denotes 2-norm. $\delta {\mathbf {c}}_n$ and $\delta {\mathbf {r}}_n$ are the sampled position and rotation respectively. We slightly abuse the notation and denote the number of samples as $N_s$ in this Section.

Monte Carlo Estimation An alternative method is to use Monte Carlo method. In particular, from the 3D points $\{\mathbf {{p}}^r_l\}_{l=1}^{N_f}$ and refined pose $\mathtt {{T}}^r$, we first calculate the ideal (i.e., no noise) observations $\{{\bar{\mathbf {u}}}_l\}_{l=1}^{N_f}$, where ${\bar{\mathbf {u}}}_l = \pi (\mathbf {{p}}^r_l, \mathtt {{T}}^r, K, D)$. A set of noisy measurements $\{{\tilde{\mathbf {u}}}_l\}_{l=1}^{N_f}$ can be simulated by ${\tilde{\mathbf {u}}}_l = \bar{\mathbf {u}}_l + {\mathbf {n}}_{\mathbf {u}}$, where ${\mathbf {n}}_{\mathbf {u}}\in {\mathcal {N}}({\mathbf {0}}, \varvec{\Sigma }_{\mathbf {u}})$. We then can compute a camera pose estimate $\mathtt {{T}}^m$ using the method in Sect. 3.3 with the simulated feature locations $\{{{\tilde{\mathbf {u}}}}_l\}_{l=1}^{N_f}$ instead of the actual ones $\{\mathbf {u}_l\}_{l=1}^{N_f}$. After repeating the above process $N_s$ times, the uncertainty of camera position $\sigma ^{c}_m$ and rotation $\sigma ^{r}_m$ can be computed as

5 $$\begin{aligned} \delta ^{c}_{m}= \text {median}\big (\{\epsilon ^{c}_n\}_{n=1}^{N_s}\big ) \quad \delta ^{r}_{m} = \text {median}\big (\{\epsilon ^{r}_n\}_{n=1}^{N_s}\big ), \end{aligned}$$

where $$\epsilon ^c_n, \epsilon ^r_n = \mathtt {{T}}^r \boxminus \mathtt {{T}}^m_n$$ [cf. (7)].

Sampling Uncertainty We also explore a sampling strategy for uncertainty estimation in this paper. In particular, for a sampling ratio *k* (e.g., $50\%$), we first randomly sample from all the 2D–3D matches (i.e., all the inliers used in (3)). We then apply LO-RANSAC to the sampled subset and solve the nonlinear optimization problem (3) using the inliers returned by LO-RANSAC to get a pose $\mathtt {{T}}^s$. The sampling and solving process is repeated multiple times (typically 50 times in our experiment), resulting in multiple pose estimates $\{\mathtt {{T}}^s_n\}_{n=1}^{N_s}$. The sampling uncertainty for the camera position $s^{c}_{k}$ and rotation $s^{r}_{k}$ are calculated as

6 $$\begin{aligned} s^{c}_k = \text {median}(\{\epsilon ^{t}_n\}_{n=1}^{N_s}) \quad s^{r}_k = \text {median}(\{\epsilon ^{r}_n\}_{n=1}^{N_s}), \end{aligned}$$

where $\epsilon ^c_n, \epsilon ^r_n = \mathtt {{T}}^r \boxminus \mathtt {{T}}^s_n$ [cf. (7)]. We calculate the sampling uncertainties for different sampling ratios.

We would like to highlight the difference between the uncertainties computed from the above methods and the absolute uncertainties. The absolute uncertainties reflect the differences between the refined poses and the *unknown* ground truth, which cannot be calculated directly. The above uncertainties, on the other hand, evaluate the variance (by approximation or computing statistics from randomized sampling) with respect to the refined poses, which are essentially the local minima in the optimization problem (3). Therefore, these uncertainties tend to be smaller than the actual uncertainties, since the local minima can hardly be the actual ground truth poses. We will further discuss how to consider these uncertainties in the context of evaluation in Sect. 4.1.

### Discussion

The method proposed in this section essentially estimates more accurate poses from some potentially inaccurate initial estimates. Yet, it can not only be used to verify and refine existing reference poses, but also to easily extend existing visual localization datasets. For example, to add more images to an existing localization dataset, one only needs to provide coarse initial poses for these images, which can be obtained by, for example, manually selecting the most similar images. This is useful especially for images with large appearance difference compared with the localization database (e.g., adding nighttime images to a localization database constructed from daytime images), where accurate poses cannot be reliably estimated using SfM directly.

## Metrics for Localization Accuracy

The reference poses generated using SfM or our method are inherently subject to inaccuracies, which complicates the evaluation process. For example, the difference between the reference pose and a pose to evaluate is no longer a meaningful metric if the actual error (i.e., the difference between the pose to evaluate and the unknown ground truth) is comparable to the uncertainty in the reference pose. Therefore, it is a common practice to set certain thresholds for the reference poses based on their uncertainties, and measure whether the poses to evaluate lie within those thresholds. Unfortunately, quantifying the uncertainties in the reference poses is a highly non-trivial task in itself. The actual uncertainties depend on various factors, such as the depth of the scene and the accuracy of the local features. In this section, we first discuss several performance metrics based on directly considering the uncertainties in pose space. We then discuss a performance metric based on the re-projection of the scene points, which removes the necessity of directly quantifying the pose uncertainty.

### Direct Pose Uncertainty-Based Measures

Direct pose uncertainty-based measures analyze the position and rotation error between the reference and estimated poses. Typically, given a reference pose $\mathtt {{T}}$ and a pose to evaluate ${\hat{\mathtt {{T}}}}$, the position and rotation error $\epsilon ^c, \epsilon ^r = \mathtt {{T}}\boxminus {\hat{\mathtt {{T}}}}$ are computed as Sattler et al. (2018):

7 $$\begin{aligned} \epsilon ^c = \Vert \mathbf {{c}}-{\hat{\mathbf {{c}}}} \Vert _2, \quad \epsilon ^r = \arccos \left( \frac{1}{2}(\text {trace}(\mathtt {R}^{-1}{\hat{\mathtt {R}}}) - 1)\right) . \end{aligned}$$

To account for the uncertainties in the reference poses, we can either use a set of fixed thresholds for all the images in a dataset or define thresholds for each image individually.

Fixed Error Thresholds We can define a set of $N_e$ increasing error thresholds $E^{\text {fixed}} = \{{\mathbf {e}}^{\text {pose}}_j\}_{j=1}^{N_e}$, where ${\mathbf {e}}_j = (c_j, r_j)$ contains both position and orientation thresholds. These thresholds apply to all the images in a dataset. A pose is said to be below a threshold ${\mathbf {e}}_j$ if $\epsilon ^c < c_j$ and $\epsilon ^r < r_j$. The overall localization accuracy is the percentages of images that are localized within these thresholds, and higher values indicate better performance. For example, the error thresholds for Aachen night time images on visuallocalization.net are 0.5/1.0/5.0 m and 2.0/5.0/10.0 ${\mathrm{deg}}$, and the localization accuracy is reported as three percentages corresponding to the these categories.

Per Image Error Thresholds Using the same thresholds for all the images in a dataset, however, has limitations. The uncertainties are image-dependent if, as in our case, the poses are calculated by minimizing the reprojection errors of 2D–3D correspondences. The position uncertainty is lower for images observing landmarks that are closer to the camera. Ideally, these uncertainties should be taken into consideration to choose the error thresholds *per image*. As shown in Sect. 3.4, there are different ways of computing the pose uncertainty for each image, which can be used as per image error thresholds. For the first order and Monte Carlo uncertainties, we can simply use (4) and (5) as thresholds. In terms of sampling uncertainties, we can choose a set of sampling uncertainties $E^{\text {sample}}_{i} = \{{\mathbf {s}}_{k}\}_{k=k_1, k_2, ...}$ as error thresholds, where ${\mathbf {s}}_k = \{s_k^{c}, s_k^{r}\}$ is the sampling uncertainty with sampling ratio *k*. For example, in our experiment, we use a set of thresholds calculated from sampling ratios of $50\%$, $30\%$ and $10\%$ respectively. However, as discussed before, the uncertainties in Sect. 3.4 tend to be lower than the (unknown) absolute uncertainties. Therefore, using these uncertainties as error thresholds tends to under-estimate the accuracy of localization algorithms (cf. Sect. 5.5).

### Indirect Pose Uncertainty-Based Measures

To avoid the need to consider the uncertainties in 6 DoF poses (which is non-trivial as seen before), we follow the literature on object pose estimation and measure pose accuracy based on reprojections (Hinterstoisser et al. 2012). More precisely, we measure the difference between the reprojection of a set of 3D points in the reference and estimated poses. Intuitively, perturbations to the camera pose will result in the changes of the reprojected 2D locations of 3D points. Therefore, we can define certain thresholds around the reprojection of the 3D points as an *indirect* measure of the pose uncertainty. A key advantage of this approach is that the error thresholds can be defined on the image plane. While we use the same thresholds for all the images, this actually results in per-image uncertainty thresholds in pose space: the same change in reprojection error will typically result in a position error that increases with increasing distance of the camera to the scene. Formally, we define the following metric:

Maximum Reprojection Difference The maximum distance between the projected points in the reference pose $\mathtt {{T}}^r_i$ and the estimated pose ${\hat{\mathtt {{T}}}}_i$ is used to measure the localization error:

8 $$\begin{aligned} r_i^{\infty } = \underset{l\in [1, N_f^i]}{\max } \Vert \pi (\mathbf {{p}}_l^r, \mathtt {{T}}_i^r) - \pi (\mathbf {{p}}_l^r, {\hat{\mathtt {{T}}}}_i) \Vert _2, \end{aligned}$$

where the intrinsics and distortion parameters are omitted for simplicity. Similar to the pose error, a set of reprojection thresholds $E^{\text {rep}}=\{e^{\text {rep}}_j\}_{j=1}^{N_e}$ are selected, and the percentages of the images with $r_i^{\infty }$ lower than these thresholds are used to indicate the overall accuracy on the dataset. We slightly abuse $N_e$ here to denote the number of error thresholds in general.

## Experimental Evaluation

To demonstrate the value of the proposed method, we first use our method to analyze the reference poses of the nighttime query images in the Aachen Day–Night dataset (Sect. 5.2). Then, we extend the dataset with new nighttime query images and generate the corresponding reference poses using our method (Sect. 5.3). We also compare our method against baseline methods of directly matching features (SIFT and D2-Net) and computing poses via SfM models. To understand the impact of the different parameters in our method, we perform an extensive ablation study regarding different learned features, different rendering techniques, and the stability of our reference poses (Sect. 5.4). Finally, we evaluate state-of-the-art localization methods on both the original and the extended Aachen Day–Night datasets based on the performance metrics discussed in Sect. 4 (Sect. 5.5).

In this paper, we focus on the Aachen Day–Night dataset (Sattler et al. 2018, 2012). This is motivated by our observation that the reference poses for the nighttime images are the least accurate reference poses among the three datasets from Sattler et al. (2018). At the same time, the dataset is becoming increasingly popular in the community, e.g., (Sarlin et al. 2019; Yang et al. 2020; Wang et al. 2020; Benbihi et al. 2019; Dusmanu et al. 2019; Brachmann and Rother 2019; Mishchuk et al. 2017; Shi et al. 2019; Cheng et al. 2019; Revaud et al. 2019; Germain et al. 2019; Sarlin et al. 2020; Zhang et al. 2019) have already been evaluated on the dataset. However, our approach is generally applicable and can be applied to other datasets as well. Note that we only consider the nighttime query images in this paper as SfM already provides accurate reference poses for the daytime queries of the Aachen Day–Night dataset. In contrast, the authors of the dataset reported in Sattler et al. (2018) that localizing the nighttime images directly against the SfM model built from daytime images resulted in highly inaccurate poses due to a lack of sufficiently many SIFT feature matches. This is due to the strong illumination changes between the nighttime and daytime images and the limited repeatability of the SIFT descriptor to such strong changes (Zhou et al. 2016). With insufficient feature matches, pose estimation using SIFT would either fail or have erroneous results due to ill-conditioned configurations (e.g., the feature matches may concentrate in a small, well illuminated region in the image, resulting in an inaccurate pose estimate). We observe similar failure cases when using SIFT to add new nighttime images to the Aachen Day–Night dataset (see Fig. 6 and the discussion in Sect. 5.3 for examples).

### Experimental Setup and Data Acquisition

Additional Data Capture To extend the Aachen Day–Night dataset, we captured another 119 nighttime images and 119 daytime images with the camera of a Nexus 5X smart phone in July 2017. The nighttime and daytime images form pairs of photos taken from very similar poses. Registering the daytime images against the reference SfM model provided by the Aachen Day–Night dataset then yields initial pose estimates for the new nighttime queries. Both the original and the newly captured nighttime images have a resolution of $1600\times 1200$ pixels (the diagonal is thus of 2000 pixels).

Scene Model Generation Our approach to refine camera poses requires an underlying 3D scene model. The Aachen Day–Night dataset provides a *reference SfM model* consisting of 4328 database images and 1.65M 3D points triangulated from 10.55M SIFT features (Sattler et al. 2018). This publicly available reference model is a sub-model of a larger *base SfM model* that was reconstructed using COLMAP (Schönberger et al. 2016). This base model also contains images from a set of videos as well as the daytime queries, resulting in a SfM model with 7604 images and 2.43M 3D points triangulated from 17.75M features. This model was registered against the original Aachen SfM model from Sattler et al. (2012) to recover the scale of the scene. The reference model was obtained by removing the sequences and query images from the base model.

We started from the base model and created an *extended SfM model*. We registered the additional daytime images and an additional image sequence^3^ against the base model while keeping the poses of the base model images fixed. The resulting model contains 12,916 images and 3,90M 3D points triangulated from 32.19M SIFT features. We used this extended base model when creating our new reference poses.

We removed all query images and the newly added sequence images from the extended base model to create an *extended reference SfM model* consisting of 6697 images and 2.32M points triangulated from 15.93M SIFT features. This model will be used to benchmark localization algorithms on our extended Aachen Day–Night dataset. We will make this new reference model publicly available, but will withhold the base models and the reference poses for the query images. Instead, we will provide an evaluation service on visuallocalization.net. The motivation behind publishing this smaller dataset is to make sure that the reference poses were computed from additional data not available to localization algorithms. The inclusion of the original sequences is necessary as some of the newly added nighttime queries depict places not covered in the original reference model.

In addition to the extended models, we also created a colored 3D mesh of the scene. We used COLMAP’s Multi-View Stereo pipeline (Schönberger et al. 2016) to obtain a dense point cloud. Screened Poisson surface reconstruction (Kazhdan and Hoppe 2013) of the point cloud then yields a colored mesh.

Rendering Our method requires rendering the scene from estimated poses. For each pose, we generate two renderings: (1) we render the MVS *mesh*, (2) we use the SfM inversion approach (*invSfM*) from Pittaluga et al. (2019) to recover an image directly from a rendering of the extended base model. We use our own implementation of invSfM. Note that we only use the CoarseNet stage and skip the VisibNet and RefineNet. We use the MVS mesh to determine which points are visible instead of VisibNet. While skipping RefineNet reduces image quality, we found the results to be of sufficient quality. Moreover, as shown in Pittaluga et al. (2019), RefineNet mostly improves the color of the rendered image with respect to the CoarseNet. Since D2-Net feature used in our method is quite robust to such changes in the view condition, we do not expect skipping RefineNet would have a large impact on the performance of our method. Figure 3 shows example renderings obtained from the mesh and invSfM.

Implementation Details If not mentioned otherwise, we extract D2-Net features (Dusmanu et al. 2019) from both rendered images. The refinement process is repeated for 5 iterations. We use single scale features since the initial pose estimates are accurate enough such that multi-scale processing is not required. To determine whether our refinement succeeded, we only accept the refined pose when there are more than 10 effective inliers^4^ found by LO-RANSAC (Lebeda et al. 2012; Sattler 2019) from the input 2D–3D matches, using the P3P solver from Kneip et al. (2011). More precisely, we subdivide each image into a $50\times 50$ grid and count at most one inlier per cell. The cell size and the inlier threshold are determined experimentally.

**Fig. 4:**
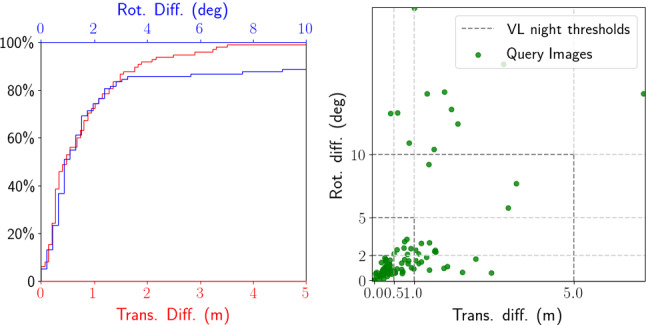
Differences between the original reference poses and the refined reference poses (our method). Left Cumulative distribution of position and rotation differences. Right Distribution of the position and rotational differences. The position and rotation thresholds (0.5/1.0/5.0 m, 2/5/10 ${\mathrm{deg}}$) used in Sattler et al. (2018) and visuallocalization.net (VL) are also shown for reference

**Table 1:** Evaluation of state-of-the-art localization methods in the original Aachen nighttime images

	Original poses	Refined poses		
	Pose error 0.5 m, 2$^\circ$/1 m,5$^\circ$/5 m,10$^\circ$	Pose error 0.5 m, 2$^\circ$/1 m, 5$^\circ$/5 m,10$^\circ$	Sampling (50%/30%/10%)	Reprojection diff. (10/20/50/100 px)
Active Search v1.1 (Sattler et al. 2018)	27.6/38.8/56.1	48.0/57.1/64.3	2.0/4.1/11.2	28.6/39.8/52.0/62.2
D2-Net (Dusmanu et al. 2019)	45.9/68.4/88.8	86.7/96.9/100.0	7.1/13.3/35.7	46.9/68.4/89.8/98.0
DELF (Noh et al. 2017)	39.8/61.2/85.7	75.5/89.8/96.9	4.1/5.1/14.3	28.6/56.1/78.6/88.8
DenseVLAD (Torii et al. 2018) + D2-Net (Dusmanu et al. 2019)	39.8/55.1/74.5	75.5/81.6/84.7	7.1/8.2/24.5	45.9/65.3/77.6/82.7
Hierarchical Localization (Sarlin et al. 2019)	42.9/62.2/76.5	77.6/87.8/88.8	7.1/9.2/24.5	41.8/65.3/78.6/85.7
NetVLAD (Arandjelović et al. 2016) + D2-Net (Dusmanu et al. 2019)	43.9/66.3/85.7	90.8/96.9/96.9	8.2/11.2/40.8	51.0/75.5/92.9/95.9
R2D2 V2 20K (Revaud et al. 2019)	46.9/66.3/88.8	90.8/99.0/100.0	8.2/14.3/36.7	51.0/70.4/92.9/95.9

We evaluate results submitted by the authors to visuallocalization.net on both the original and our refined poses. We compare the methods based on the Pose Error, i.e., the percentage of queries localized within fixed error thresholds of the reference poses. As can be seen, our more accurate reference poses yield a better measure of pose accuracy. For our poses, we also report results for two additional metric: the percentage of queries localized within sampling-based thresholds (Sampling) of the reference poses (cf. Sect. 4.1) and the percentage of queries with maximum reprojection errors within given error thresholds in pixels (Reprojection Diff.) (cf. Sect. 4.2)

### Refining the Original Aachen Nighttime Poses

In a first experiment, we analyze the accuracy of the reference poses for the 98 original nighttime queries of the Aachen Day–Night dataset. We show that the original reference poses are inaccurate and that our refinement approach considerably improves the pose accuracy.

Our approach used the original poses for initialization. For 3 out of the 98 images, our method failed to find sufficiently many 2D–3D matches, mostly due to an incomplete mesh (see Sect. 5.4). For the failure cases, we simply kept the existing reference poses.

Qualitative Evaluation Figure 3 visually compares the original reference poses with our refined poses. As can be seen, the existing reference poses, obtained from manual annotated 2D–3D matches, can be rather inaccurate. In contrast, our method generates reference poses such that the rendering from the refined pose is visually consistent with the actual image. Thus, features matching between the real and rendered images are found at the same positions (up to noise), as can be see from the (short) green lines. Figure 3 shows selected examples where the original reference poses were rather inaccurate. Visual comparison between the original and our refined poses showed that our approach consistently produced more accurate poses for all nighttime queries.

It is also worth noting that D2-Net features can provide robust matches even though the rendered images (using a model reconstructed from daytime imagery) are visually very different from the actual images and contain non-trivial rendering artifacts.

Quantitative Evaluation To quantify the differences between the original and our reference poses, we computed the differences in camera position and orientation [see (7)]. Figure 4 shows the results of this comparison. It can be seen that there exists a non-trivial discrepancy between the original and refined reference poses.

Sattler et al. (2018) measures localization accuracy by the percentage of nighttime query poses estimated within (0.5 m, 2 ${\mathrm{deg}}$), (1 m, 5 ${\mathrm{deg}}$), and (5 m, 10 ${\mathrm{deg}}$) of the reference poses. These thresholds are also shown in Fig. 4. As can be seen, the differences between the original and refined poses fall outside of the largest error threshold for 11 images ($\sim 11.2\%$ of all the nighttime queries). Interestingly, the best results reported on visuallocalization.net register 88.8% of the nighttime queries within 5 m and 10 ${\mathrm{deg}}$. Thus, state-of-the-art methods might actually be more accurate than the reference poses.

Finally, Table 1 evaluates several state-of-the-art localization methods using the existing and refined reference poses. As can be seen, the accuracy of the localization methods is indeed (significantly) under-estimated by the existing reference poses of the nighttime images in the Aachen Day–Night dataset. In contrast, our reference poses allow us to measure localization performance more accurately. Table 1 also provides results for additional evaluation measures for our new reference poses, which will be discussed in Sect. 5.5. Note that the improvement reported here is particular to the nighttime images in the Aachen Day–Night dataset. The result on a different dataset will depend on the quality of the existing reference poses in the dataset.

Summary Our results clearly show that our new reference poses are more accurate than the original poses. We will integrate our new poses in the visuallocalization.net online benchmark, allowing us to easily update all results on the website.

**Fig. 5:**
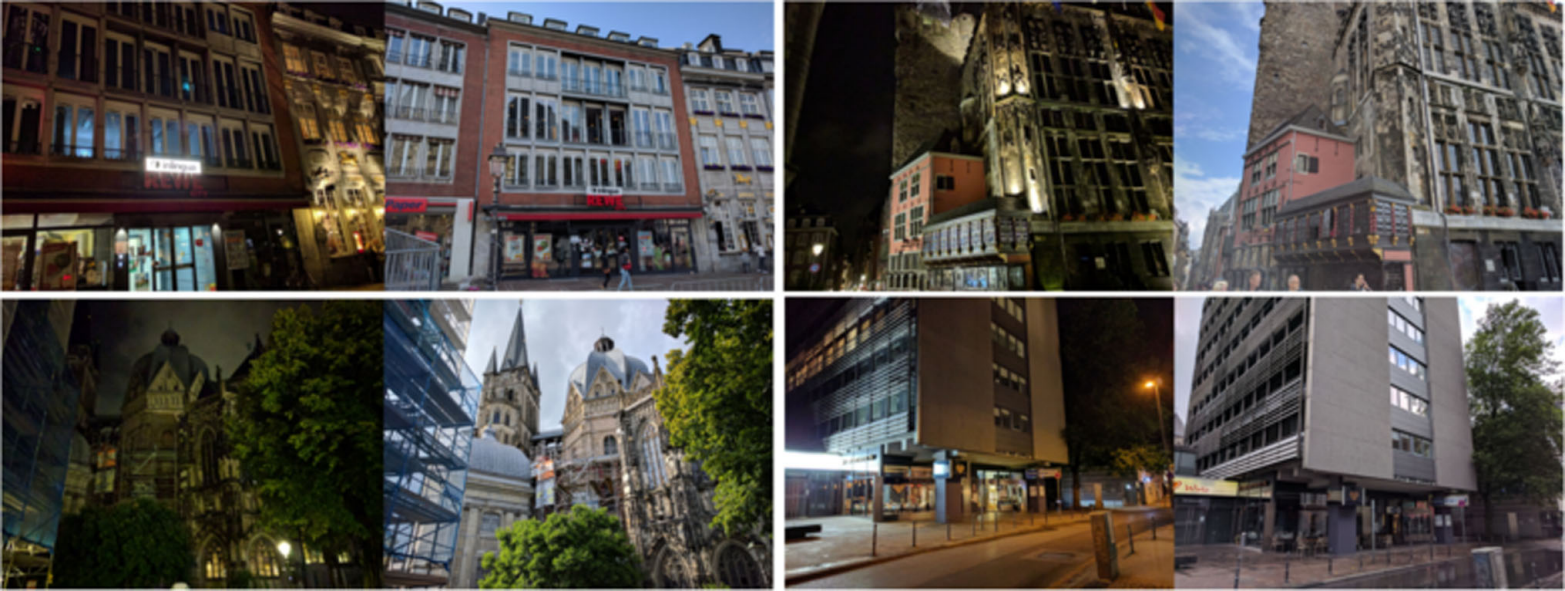
Pairs of day–night images taken from similar poses. We obtain reference poses for the daytime images via SfM. The resulting poses are used to initialize our approach for generating poses for the nighttime images. For the SIFT and D2-Net registration baselines, an additional 20 daytime images that overlap with these images are selected from the base model for the daytime image in each pair

### Extending the Aachen Day–Night Dataset

Our approach is capable of estimating an accurate pose from a coarse initialization. Besides verifying and refining existing reference poses, our approach can also be used for generating reference poses for new images. In the next experiment, we thus extend the Aachen Day–Night dataset by additional nighttime queries. We compare our reference poses with two registration baselines using SIFT and D2-Net features, respectively.

Reference Pose Generation As shown in Fig. 5, we captured a daytime photo from a similar pose for each the 119 new nighttime images. The poses of these daytime images in the extended base model, obtained via SfM, then provide initial pose estimates for the nighttime queries that are subsequently refined by our approach. We excluded images for which our method resulted in less than 10 effective inliers to avoid unreliable reference poses. This results in reference poses for 93 out of the 119 images.

We compare our method with two baselines using SIFT and D2-Net features, respectively. Both baselines match features between the 93 new nighttime queries and a small set of images in the extended base SfM model. For a nighttime query, this set includes the corresponding daytime image ${\mathcal {I}}_D$ as well as the 20 images in the extended base model that share the most 3D points with ${\mathcal {I}}_D$. 2D–2D matches between the nighttime image and the daytime photos in the set then yield a set of 2D–3D correspondences based on the 3D points visible in the latter. COLMAP’s image registration pipeline was then used to obtain the camera pose based on these matches. Note that for D2-Net features, we re-triangulated the extended base 3D model before day–night feature matching.

**Fig. 6:**
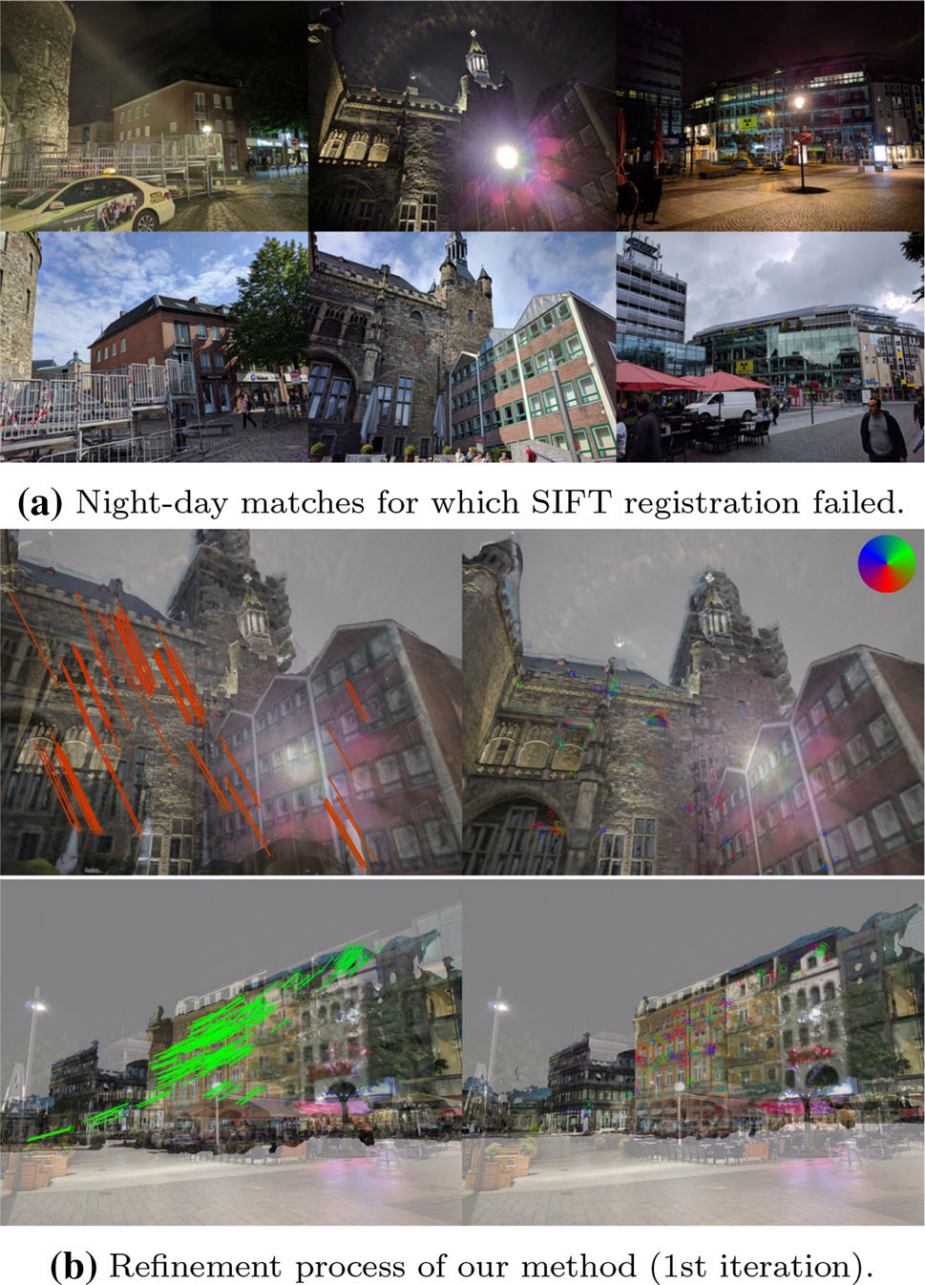
Typical failure cases of the SIFT registration baseline. Top nighttime images where SIFT registration failed and the corresponding daytime images; Bottom Visualization of the first iteration of our method (left: initial pose; right: refined pose). The differences between D2-Net features and the projection of the matching 3D points are color coded according to the direction in the image plane (cf. legend in the top-right)

Robustness Both the D2-Net baseline and our method are able to consistently estimate poses for challenging images for which the SIFT baseline fails. Figure 6 shows such failure cases of SIFT. In each of the shown cases, there is a strong light source in the scene, causing significant appearance differences between the day and nighttime images. SIFT is not able to deal with these strong changes. In contrast, our method, as well as the D2-Net baseline, which relies on high level learned features, are able to handle these cases (cf. Fig. 6b).

Sattler et al. (2018) reported that the reference poses obtained via SfM and SIFT were unreliable. Interestingly, we observe the opposite for many images in our experiments. We attribute this to the inclusion of the corresponding daytime images: as shown in Torii et al. (2018), SIFT features better handle day–night changes under small viewpoint changes. Note that daytime images taken from very similar poses are not available for the original nighttime queries.

**Fig. 7:**
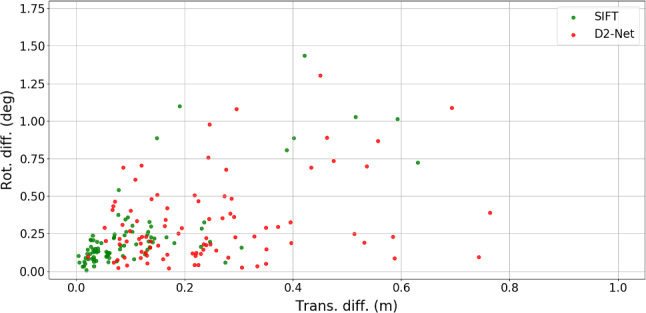
Distribution of the pose difference between our method and the two registration baselines

Quantitative Evaluation Excluding the failure cases, we computed the pose differences between our method and two baselines. The results of this comparison are shown in Fig. 7. Interestingly, the poses from our method and the SIFT registration are very consistent. For the majority of the images, the pose difference is below 0.2 m and 0.5 ${\mathrm{deg}}$. In contrast, we observe much larger difference between our poses and the D2-Net registration baseline. As there is no external reference poses that can be used to calculate the absolute pose accuracy, we resort to visual inspection based on the renderings.

**Fig. 8:**
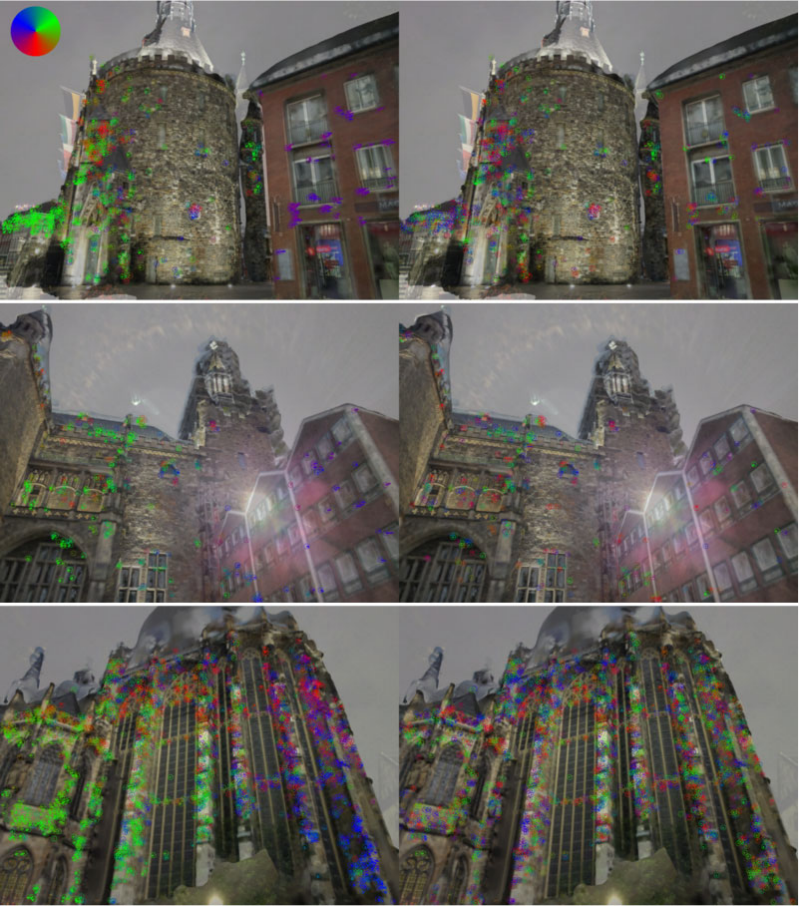
Comparing the D2-Net baseline against our refinement. Left overlay of real photos and images rendered with D2-Net poses. D2-Net features in the rendered images are connected to the matching locations in the real images (circles), and the color indicates the direction of the feature location differences in the two images (see legend in the top-left). Right corresponding visualization using poses obtained by one iteration of our method (initialized with D2-Net poses). The patterns of the feature directions in the left images indicate the inaccuracy in the poses from D2-Net registration, which are corrected with our method (right images)

Visual Inspection Figure 8 analyses example poses obtained by the D2-Net baseline. Besides overlaying the real and rendered images, we also show D2-Net features matches between the two. For each match, we compute the 2D offset between the feature positions in the real and the rendered view. Following Schops et al. (2019), we color-code the features based on the directions of these 2D offsets. As argued in Schops et al. (2019), these directions should be randomly distributed for accurate pose estimates. Patterns of similar direction in the same region of an image indicate a shift between the two images and thus pose errors.

The D2-Net poses in Fig. 8 are visually more accurate than those in Figures 3 and 6b. Still, we observe clear patterns in the distribution of the directions (e.g., the concentration of green color on one side and purple on the other), which indicates inaccuracies in the poses of the D2-Net baseline. We further used one iteration of our method to refine the D2-Net poses. As can be seen in Fig. 8, the refinement improves the distribution of directions. We conclude that our approach is able to provide more accurate poses than the D2-Net baseline.

**Fig. 9:**
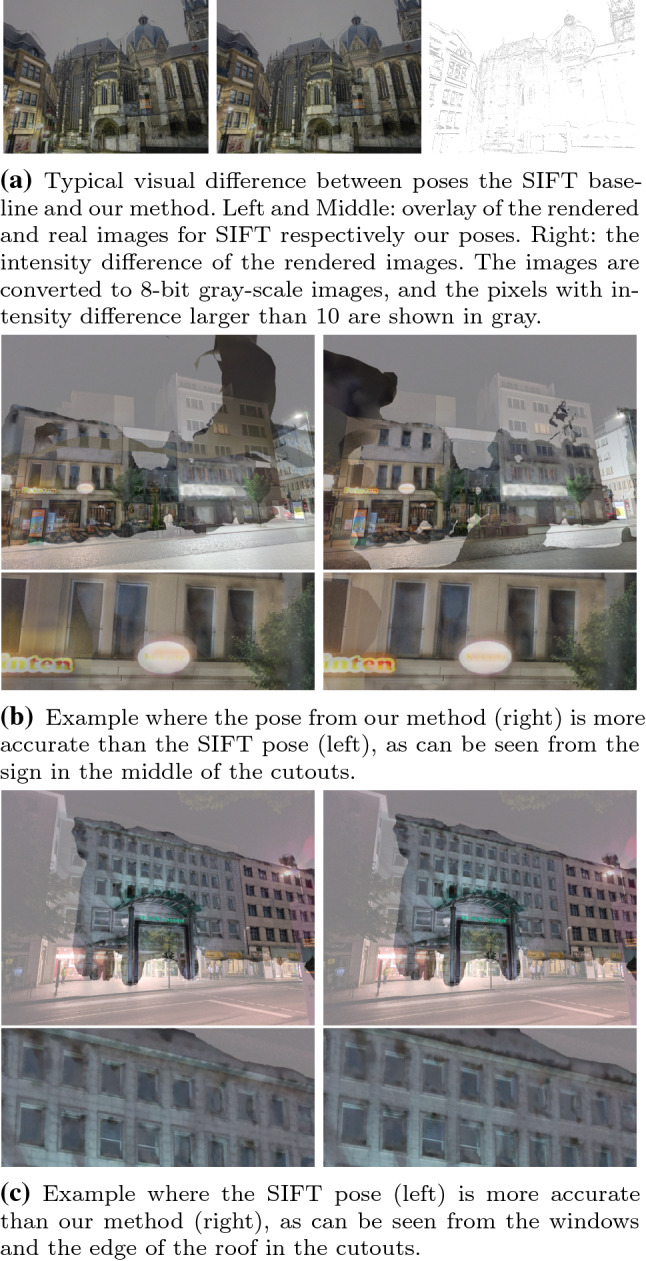
Visual comparison of images rendered from the poses obtained by our method and the SIFT baseline

As can be seen from Fig. 7, the pose differences between our approach and the SIFT baseline are significantly smaller than the differences between our approach and D2-Net. Unlike for D2-Net poses, we did not see strong feature direction patterns for the SIFT poses. We therefore omit the corresponding visualizations. We observe that if the SIFT baseline is able to estimate a pose it is usually visually similar to the pose obtained with our approach (cf. Fig. 9a). There are images where the poses from our method seem to be visually more accurate than the SIFT registration and vice versa (shown in Fig. 9b, c, respectively). Yet, overall there are only 7 out of the 93 new nighttime queries for which we consider the SIFT poses to be visually more accurate than the poses provided by our method. For these images, we use the SIFT poses as reference poses. At the same time, SIFT failed to provide poses for 5 of the nighttime images due to a lack of sufficient matches.

Discussion and Summary It is interesting to see that SIFT poses are not necessarily more accurate than our poses. SIFT features are much more accurately localized in images than D2-Net features (Dusmanu et al. 2019). Thus, one might have expected that a few accurately localized SIFT matches are better than many less accurately localized D2-Net matches. Yet, finding more matches with D2-Net between the renderings and the real images seems to compensate for the inaccuracy of the D2-Net feature detections.

For the newly acquired nighttime images, we observe that our approach performs similar to SIFT in terms of accuracy. In this case, SIFT benefits from daytime images taken from similar viewpoints. As evident from the failure cases of SIFT on both the original and new queries, our approach is more robust than the SIFT baseline. As a result, our approach is better suited to for reference pose generation for datasets that benchmark long-term visual localization algorithms.

Compared with the D2-Net baseline, the poses resulting from our method are more accurate. The main difference between the D2-Net baseline and our approach is the use of rendered images. The results thus validate our choice to iteratively render the scene from the current pose estimate and match features against the rendering. Moreover, as seen from the analysis of the D2-Net baseline, the ability of our method to verify and refine existing poses is also valuable when it is combined with other approaches.

### Ablation Study

Next, we present ablation studies to analyze our proposed approach. We first obtain an estimate for the stability of our reference poses. Next, we determine the impact of using different features and rendering techniques, which are the two key ingredients in our method. Finally, we show failure cases of our method.

Pose Stability To provide a quantitative measure of the uncertainties/stability of the reference poses obtained with our method, we compute the sampling uncertainties as described in Sect. 3.4 for both the original and additional nighttime images: we randomly sample a percentage of 2D–3D matches from the inliers used to estimate the reference poses. This sample is then used to obtain another pose estimate. The differences between these new and our poses provide a measure for the stability of the minima found by our approach.

We used three sampling rates that use 90%, 50%, and 10% of the inliers, respectively. For each rate, we drew 50 random samples and report the median position and orientation differences. In addition, since our method uses different rendering techniques and is an iterative process, we also computed the following for comparison:

- *Compare-InvSfM* the differences between the refined poses using both types of rendered images and using InvSfM only;
- *Compare-Mesh* the differences between the refined poses using both types of rendered images and mesh rendering only;
- *Compare-Prev-Iter* the pose differences between the two last iterations of our refinement process.

**Fig. 10:**
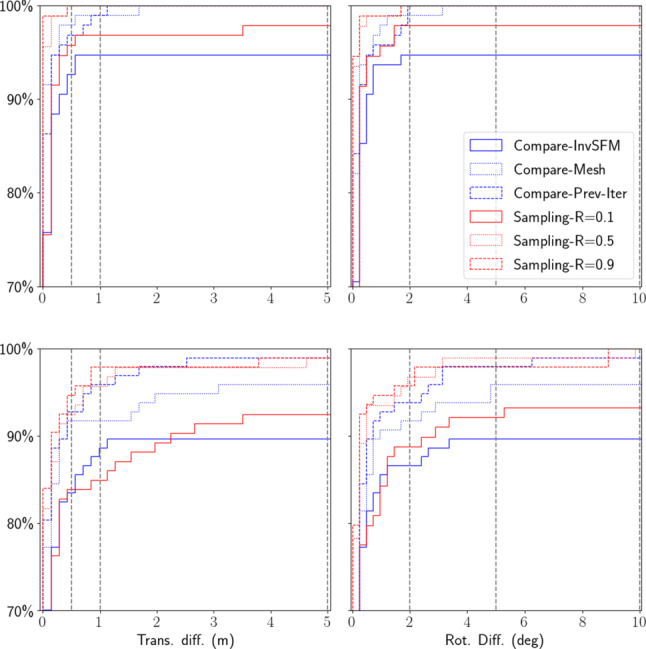
Different uncertainties for the original (top) and additional (bottom) Aachen Night images. The vertical dash lines corresponds to the error thresholds proposed in Sattler et al. (2018) and used by the online benchmark

**Fig. 11:**
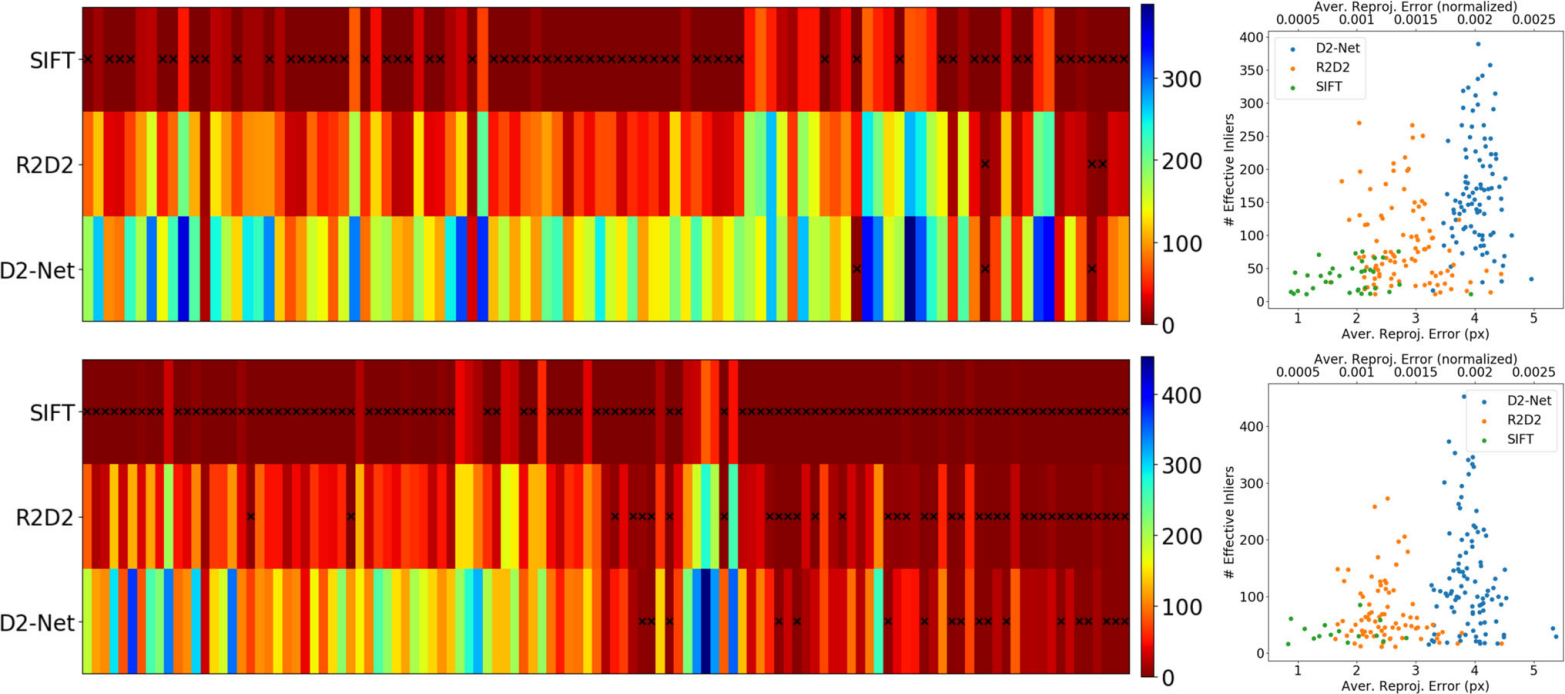
Effect of using different features in our method. Left the number of effective inliers for each image. Each block along the horizontal axis corresponds to one image. A black cross indicates there are less than 10 effective inliers, i.e., the pose is likely not reliable. Right the number of effective inliers and the mean reprojection error (after nonlinear optimization) for different features. Failure cases (i.e., the black crosses) are excluded. The normalized reprojection error is normalized by the image diagonal length. The top row shows the result for the original Aachen nighttime images, and the bottom for additional images

The results of our comparisons are shown in Fig. 10. For the original images, more than $90\%$ of the images are below the finest error threshold (0.5 m, 2 ${\mathrm{deg}}$) of the visual localization benchmark, independently of which sampling rate and rendering is used. For the additional images, the uncertainties are higher. Still, more than $80\%$ of the images fall in that threshold as well. The fact that the uncertainties of the additional images are overall higher than the original images indicates that the newly added images might be more challenging. Regarding the different rendering techniques, images rendered using the MVS mesh seem to provide more information for the final refined poses, as *Compare-Mesh* shows less uncertainty than *Compare-InvSfM*.

While it is difficult to quantify the absolute uncertainties, the uncertainties shown in Fig. 10 indicate that the reference poses generated using our method are at least stable solutions considering the available 2D–3D matches. This can be seen from the fact that even using as little as 10% of the available inlier matches leads to very similar pose estimates for nearly all images.

Different Features Instead of using D2-Net features, we also used SIFT and R2D2 (Revaud et al. 2019) features to obtain matches between the rendered and real images.

Figure 11 compares the results obtained with different types of features. As can be seen, SIFT failed to find enough matches in most cases for both the original and additional night images. This is not surprising considering SIFT relies on low-level image statistics, which are strongly impacted by imperfections in the MVS model and the invSfM rendering process. In contrast, both D2-Net and R2D2 features were able to find enough matches for most of the original Aachen night images. The success rate for both features drops on the additional Aachen night images, where the D2-Net feature performed better. Plotting the reprojection error (after nonlinear optimization) against the number of effective inliers, we observe a clear trend across different features: D2-Net recovers the most matches, followed by R2D2 and SIFT; while SIFT features were most accurately localized in the images, D2-Net has the largest reprojection errors.

**Fig. 12:**
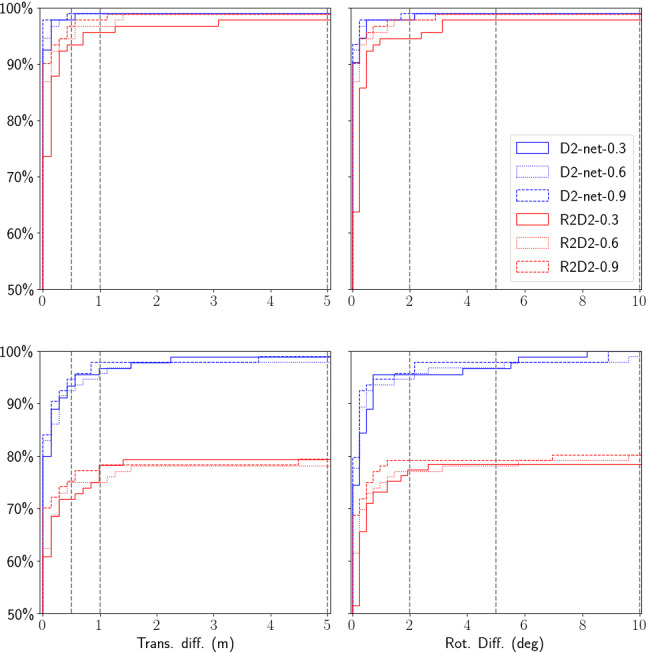
Sampling uncertainties of the D2-Net and R2D2 poses for the original (top) and additional (bottom) Aachen night images. Median position and orientation errors over 50 random samples are shown

To see how the number of effective inliers and reprojection error translates to the quality of the refined poses, we further computed the sampling uncertainties for D2-Net and R2D2, shown in Fig. 12. We excluded SIFT since it failed for most of the images. It can be seen that the refined poses from D2-Net features are more stable than the R2D2 poses for both the original and additional images.

The results validate our choice of using D2-Net features to match between real and rendered images as they better handle imperfections in the renderings.

**Fig. 13:**
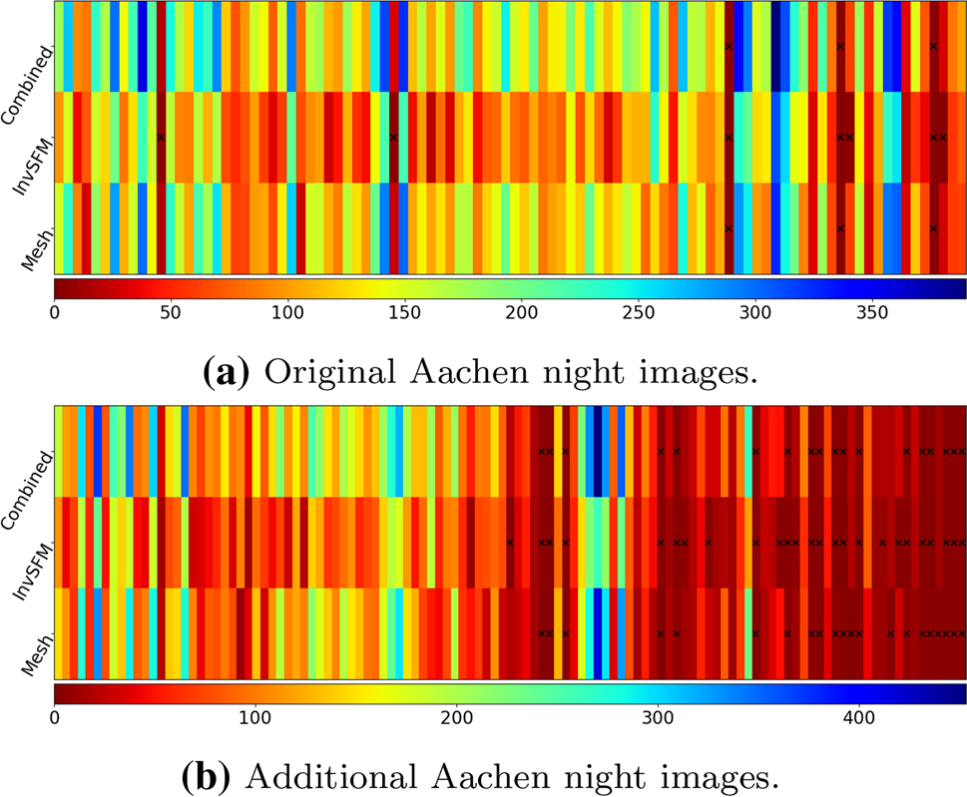
The number of effective inliers for D2-Net features when different rendering techniques are used. The visualization is the same as Fig. 11

Different Rendering Techniques The experiments presented so far used both rendering types (using MVS mesh and the invSfM process). Next, we compare using both types against using only one of the two using the number of effective inliers.

As can be seen in Fig. 13, using renderings based on the MVS mesh in general resulted in more effective inliers compared to using invSfM for rendering. Accordingly, there are more images where our method could find sufficient effective inliers in the images rendered from mesh. This is also consistent with our results in Fig. 10, which show that the poses based only on mesh rendering are more accurate than those obtained using only invSfM. Yet, there are a few cases where mesh rendering fails while invSfm rendering succeeds. The corresponding nighttime images show parts of the model that are only sparsely covered by images and where the MVS reconstruction is thus incomplete. The invSfM process seems to be more stable for such cases.

Combining the 2D–3D matches obtained from both types of renderings increases the number of effective inliers. Note that the effective inlier count selects at most one inlier for each 50 pixels by 50 pixels region in an image. A higher effective inlier count thus indicates that the matches found by the two rendering types are somewhat complimentary as matches are found in different image regions. Moreover, there are a few cases (right part of Fig. 13b) for which using both rendering types is necessary to obtain sufficiently many inliers.

The results validate our choice of using both rendering techniques as they are (partially) complimentary.

**Fig. 14:**
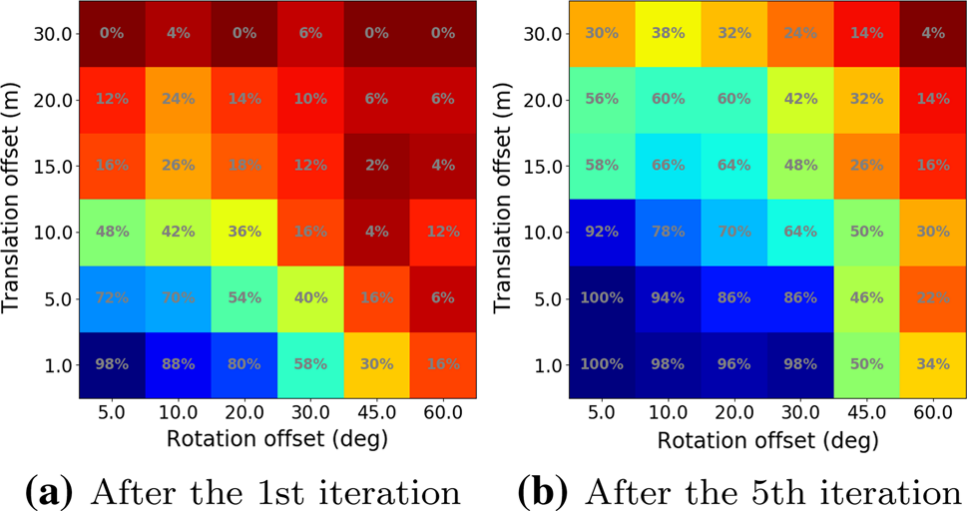
Success rates of our method in the presence of different initial error levels on the original Aachen nighttime images using D2-Net features. Each cell corresponds to the success rate of 50 trials of pose refinement using perturbed poses as input. A perturbed pose was generated by adding both random rotation and position offsets of certain magnitudes, as indicated on the *x* and *y* axes respectively, to a refined pose. The refinement of a pose is considered successful if the pose error after refinement is within 0.25 m and 1.0 deg

Sensitivity to Initialization Our approach requires an initial pose estimate as input to the iterative refinement. Naturally, our approach will fail if the initial pose estimate is not accurate enough. To determine the sensitivity of our approach to the initial pose error, we randomly perturbed the refined poses by translations and rotations of different magnitudes and used the perturbed poses as input to our method. We then measured the sensitivity of our method to the initial pose error by the success rates at different perturbation levels, and the refinement is considered successful if the manually added error can be reduced to under 0.25 m and 1.0 deg, which is half of the smallest fixed error threshold used in our evaluation (cf. Sect. 5.5). The experiment was performed on the original Aachen nighttime images using D2-Net features. As can be seen in Fig. 14b, the success rate of our method is more than $50\%$ for initial pose errors up to 30 deg and 10 m. The success rate is more than $90\%$ for disturbances within 10 deg and 5 m. Moreover, the increase of the success rates from the 1st iteration (Fig. 14a) to the 5th iteration (Fig. 14b) indicates the necessity of performing the refinement iteratively multiple times. Therefore, our method is quite robust to the errors in initial poses and can be potentially used with systems that provide less accurate localization information (e.g., GPS) to get more accurate poses automatically.

**Fig. 15:**
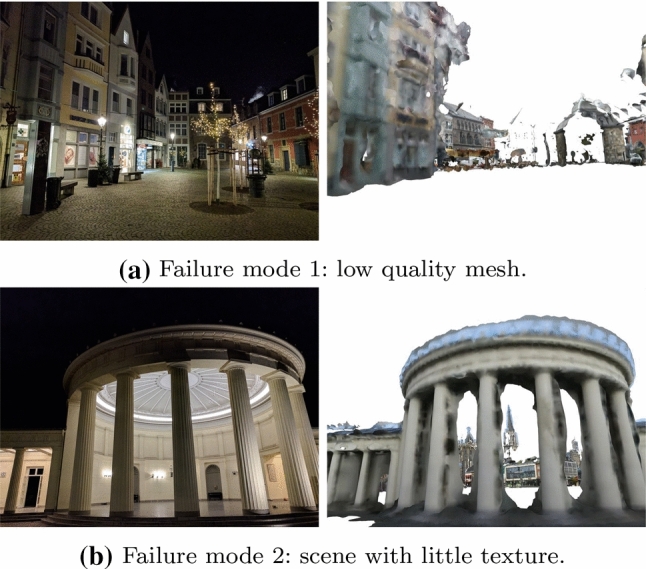
Typical failure cases of our method. Left: real nighttime images; Right: MVS mesh renderings from the initial pose

Failure Cases Figure 15 shows examples of two typical failure cases of our method. The first failure mode is when the nighttime image was taken in a part of the scene where the MVS mesh is of low quality, e.g., parts of the surface have not been reconstructed (cf. Fig. 15a). This could be overcome by using a more complete/higher quality mesh of the scene, but might require additional data capture. The second failure mode is caused by weakly textured scenes (cf. Fig. 15b). In the shown example, the rendered image is of reasonable quality visually. However, due to the lack of texture, our method failed to find enough matches between the rendered image and the real night image. Using contour edges as an additional feature type could help avoid this failure mode. However, edges are also typically harder to match than local features. Furthermore, care would need to be taken to handle protruding regions in the MVS model.

**Table 2:** Localization accuracy using different metrics on the extended Aachen Day–Night dataset

	Original night images			All night images		
	Pose error 0.5 m, 2$^\circ$/1 m, 5$^\circ$/5 m, 10$^\circ$	Sampling (50%/30%/10%)	Reprojection diff. (10/20/50/100 px)	Pose error 0.5 m, 2$^\circ$/1 m, 5$^\circ$/5 m, 10$^\circ$	Sampling (50%/30%/10%)	Reprojection diff. (10/20/50/100 px)
D2-Net	90.8/98.0/98.0	11.2/19.4/43.9	56.1/80.6/92.9/95.9	90.6/97.4/97.9	6.3/11.0/30.9	36.1/73.8/91.1/96.9
R2D2-20k	90.8/95.9/95.9	7.1/11.2/38.8	54.1/76.5/89.8/93.9	88.5/94.8/96.3	5.2/7.9/29.8	40.8/72.8/91.6/94.8
R2D2-40k	91.8/98.0/98.0	7.1/13.3/44.9	56.1/76.5/92.9/95.9	88.5/95.3/97.9	5.8/8.9/33.0	41.9/73.3/91.6/95.8

We compare the methods based on the Pose Error, i.e., the percentage of queries localized within fixed error thresholds of the reference poses. As can be seen, our more accurate reference poses yield a better measure of pose accuracy. For our poses, we also report results for two additional metric: the percentage of queries localized within sampling-based thresholds (Sampling) of the reference poses (cf. Sect. 4.1) and the percentage of queries with maximum reprojection errors within given error thresholds in pixels (Reprojection Diff.) (cf. Sect. 4.2)

### Evaluation of State-of-the-Art Methods

Table 1 evaluates published state-of-the-art localization methods using our new reference poses for the original nighttime images. The results were obtained by re-evaluating poses submitted to visuallocalization.net.^5^ In the following, we present results for state-of-the-art methods on our new extended Aachen Day–Night dataset. Note that the extended dataset uses a larger reference SfM model than the original one and we thus cannot use results from the benchmark website.

Given that D2-Net and R2D2 features achieve state-of-the-art results in Table 1, we use two image retrieval-based approaches based on these features in our evaluation. Both approaches first re-triangulate the reference SfM model with feature matches between the reference images found by D2-Net respectively R2D2. Next, NetVLAD (Arandjelović et al. 2016) is used to retrieve the 20 most similar reference image for each nighttime query. Feature matches between each query and its retrieved image yield a set of 2D–3D matches via the 3D points visible in the reference images. These 2D–3D matches are used for pose estimation against the reference model inside COLMAP.^6^ For R2D2, we provide results for two variants that use at most 20k (R2D2-20k) and 40k (R2D2-40k) features per image respectively.

Table 2 shows the results of our experiments using the evaluation measures discussed in Sect. 4. Similarly, Table 1 also shows results for all metrics for our new reference poses. Overall, the accuracy is lower when considering all nighttime queries compared to only focusing on the original night images, independent of the metric used. This indicates the newly added images might be more challenging. In the following, we discuss the results per evaluation metric.

Pose Error with Fixed Thresholds We consider the three fixed error thresholds used in Sattler et al. (2018) and on the benchmark website, i.e., (0.5 m, 2 ${\mathrm{deg}}$), (1 m, 5 ${\mathrm{deg}}$), and (5 m, 10 ${\mathrm{deg}}$). Based on the this metric, the performance on the original and extended Aachen dataset seems saturated for certain algorithms (e.g., D2-Net and R2D2). However, these thresholds were originally chosen to take the uncertainties in the original nighttime reference poses into account. As shown in our previous experiments, our new reference poses are significantly more accurate. As such, using rather loose thresholds could lead to an overestimate in the localization accuracy. Furthermore, as discussed in Sect. 4, using the same thresholds for all images does not take into account that the uncertainty in the pose depends on the distance of the camera to the scene.

**Fig. 16:**
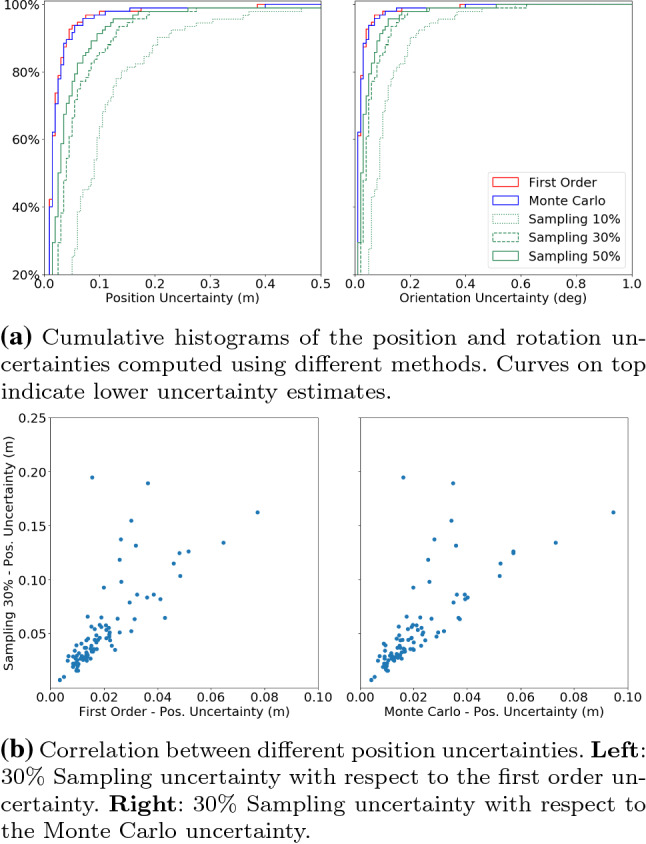
Comparison of the different uncertainty definitions provided in Sect. 3.4 on the original Aachen nighttime images using the refined poses

Per Image Error Thresholds The second metric aims at computing error thresholds on the camera pose per image. We first show the results using the sampling uncertainties (6) as error thresholds and then discuss the first order and Monte Carlo uncertainties (4) and (5). For each reference pose, we randomly sampled set containing $10\%$, $30\%$ and $50\%$ of the inliers of our method. For each sampling percentage, we drew 50 samples and computed the median position and orientation difference between the poses obtained from the samples and the reference poses. These median differences were then used as the error thresholds. As can be seen from Table 1 and Table 2, the sampling uncertainties tend to under-estimate the localization performance of the different methods. This is due to the fact that our reference poses are rather stable under using a subset of the inlier matches (cf. Fig. 12). The sampling uncertainties reflect the stability of the local minimum reached in the refinement process, rather than the absolute uncertainties. Thus, this metric should not be used to evaluate localization performance.

As for the first order and Monte Carlo uncertainties, we found that they tend to be lower than the sampling uncertainties. As an example, a comparison of different uncertainties on the original Aachen nighttime images (using the refined pose) is shown in Fig. 16. From Fig. 16a, we can see that the uncertainties estimated by first order and Monte Carlo methods are in general lower than the sampling uncertainties, even for the highest sampling ratio of $50\%$. We also inspected the correlation between different uncertainties and visualized an example of the position uncertainties in Fig. 16b. It can be seen that different uncertainties show similar trend across images, but the sampling uncertainties tend to be higher than the uncertainties computed from the first order and Monte Carlo methods (notice different axis scales). Therefore, using the first order and Monte Carlo uncertainties as error thresholds will under-estimate the localization performance as well (even worse than the sampling uncertainties) and thus should not be used as accuracy metrics.

Maximum Reprojection Difference Our reference poses are obtained by minimizing a reprojection error in image space, rather than an error in camera pose space. Thus, evaluating localization algorithms based on the quality of their reprojections seems a natural metric, especially if these algorithms compute poses by minimizing an image space error.

For each 3D point in the inlier 2D–3D matches of the reference poses, we compute a reprojection difference between the reference and an estimate pose. For each image, we report the maximum difference and we compute the percentages of images that have a maximum reprojection difference below 10, 20, 50 and 100 pixels. Since all nighttime images have a resolution of 1600$\times$1200 pixels, these thresholds correspond to 0.5%, 1%, 2.5%, and 5% of the image diagonal.

Comparing the results with the pose error metric using fixed thresholds, we can see that although the top performing algorithms achieve approximately 90% in the finest pose error category, they only have 70–80% of all the images that were localized within 20 pixel according to the maximum reprojection difference. Even less images are localized within 10 pixels. Since the accuracy of local features are typically below 5 pixel [cf. Fig. 11(right)], this indicates that there is still much room for improvement on our extended version of the Aachen Day–Night dataset. As such, we believe that the maximum reprojection error metric should be the metric of choice for this dataset.

## Conclusion

In this paper, we have considered the problem of creating reference camera poses for long-term visual localization benchmark datasets. In this setting, classical features often struggle to obtain matches between images taken under strongly differing conditions. At the same time, human annotations are both time-consuming to generate and not necessarily highly accurate. Thus, we have presented an approach for refining reference poses based view synthesis and learned features that allow robust feature matching between real and rendered images. In addition, we have discussed multiple metrics for evaluating localization performance.

The main contribution of this paper is an extensive set of experiments. We have shown that the original nighttime reference poses of the Aachen Day–Night dataset are rather inaccurate. As a result, the localization accuracy of state-of-the-art methods is currently drastically under-estimate. Using our approach, we have created a more accurate set of reference poses. We will integrate these poses into the online evaluation service provided at visuallocalization.net as to provide better evaluations to the community. We also used our approach to create an extended version of the Aachen Day–Night dataset and showed that this dataset offers room for improvement. We will make the dataset available on the benchmark website. Furthermore, we will release the code for our approach as to allow other researchers to more easily build localization benchmarks.

One disadvantage of our approach is its rather slow run-time, taking about 10–20 s per iteration for a single image, where most of the time is spend for rendering and especially for the SfM inversion process. This is not an issue when creating reference poses for a benchmark, as these calculations only need to be done once and can be done offline. At the same time, our approach can be used as a post-processing step for any visual localization algorithm. An interesting research question is whether more efficient rendering techniques can be used to improve its run-time to a degree that enables online operation.

## Footnotes

1 See also visuallocalization.net/benchmark/.

2 In solving (3), iterative methods (e.g., Gauss–Newton) are usually used, where $\mathtt {{J}}_{l}$ is actually the Jacobian with respect to a small perturbation around the current estimate.

3 Using one of the original videos and extracting images at a higher frame rate.

4 The effective inlier count takes the spatial distribution of the matches in the image into account. It has been shown to be a better measure than the raw inlier count (Irschara et al. 2009).

5 There results available at visuallocalization.net for methods that outperform the approaches used in Table 1. For our experiments, we limited ourselves to methods that have been published in peer reviewed conferences and journals. Updated results for the other methods will be available on the benchmark website once we update the reference poses.

6 Based on code available at https://github.com/tsattler/visuallocalizationbenchmark.

## References

[CR1] Aiger, D., Kaplan, H., Kokiopoulou, E., Sharir, M., & Zeisl, B. (2019). General techniques for approximate incidences and their application to the camera posing problem. In *35th international symposium on computational geometry (SoCG 2019)*.

[CR2] Albl, C., Kukelova, Z., & Pajdla, T. (2016). Rolling shutter absolute pose problem with known vertical direction. In *CVPR*.

[CR3] Alcantarilla, P. F., Ni, K., Bergasa, L. M., & Dellaert, F. (2011). Visibility learning in large-scale urban environment. In *ICRA*.

[CR4] Arandjelović, R., & Zisserman, A. (2014). Visual vocabulary with a semantic twist. In *ACCV*.

[CR5] Arandjelović, R., Gronat, P., Torii, A., Pajdla, T.,& Sivic, J. (2016). NetVLAD: CNN architecture for weakly supervised place recognition. In *CVPR*. 10.1109/TPAMI.2017.2711011 28622667

[CR6] Armagan, A., Hirzer, M., Roth, P. M., & Lepetit, V. (2017) Learning to align semantic segmentation and 2.5D maps for geolocalization. In *IEEE conference on computer visual pattern recogintion (CVPR)* (pp. 4590–4597).

[CR7] Aubry M, Russell BC, Sivic J (2014). Painting-to-3D model alignment via discriminative visual elements. ACM Transactions on Graphics (TOG).

[CR8] Badino, H., Huber, D., & Kanade, T. (2011). Visual topometric localization. In *Intelligent vehicles symposium (IV)* (pp. 794–799).

[CR9] Balntas, V., Frost, D., Kouskouridas, R., Barroso-Laguna, A., Talattof, A., Heijnen, H., et al. (2019). SILDa: scape imperial localisation dataset. https://www.visuallocalization.net/datasets/.

[CR10] Balntas, V., Li, S., & Prisacariu, V. (2018, September). RelocNet: Continuous metric learning relocalisation using neural nets. In *The European conference on computer vision (ECCV)*.

[CR11] Balntas, V., Riba, E., Ponsa, D., & Mikolajczyk, K. (2016). Learning local feature descriptors with triplets and shallow convolutional neural networks. In *BMVC*.

[CR12] Bay H, Ess A, Tuytelaars T, Gool V (2008). Speeded-up robust features (SURF). Comput. Vis. Image Underst..

[CR13] Benbihi, A., Geist, M., & Pradalier, C. (2019). ELF: embedded localisation of features in pre-trained CNN. In *IEEE international conference on computer vision (ICCV)*.

[CR14] Brachmann, E., & Rother, C. (2018). Learning less is more—6D camera localization via 3D surface regression. In *CVPR*.

[CR15] Brachmann, E., & Rother, C. (2019). Expert sample consensus applied to camera re-localization. In *ICCV*.

[CR16] Brachmann, E., Krull, A., Nowozin, S., Shotton, J., Michel, F., Gumhold, S., & Rother, C. (2017). DSAC—differentiable RANSAC for camera localization. In *CVPR*.

[CR17] Brachmann, E., & Rother, C. (2020). Visual camera re-localization from RGB and RGB-D images using DSAC. arXiv:2002.12324. 10.1109/TPAMI.2021.3070754 33798073

[CR18] Brahmbhatt, S., Gu, J., Kim, K., Hays, J., Kautz, J. (2018). Geometry-aware learning of maps for camera localization. In *CVPR*.

[CR19] Brown, M., Hua, G.,& Winder, S. (2011). Discriminative learning of local image descriptors. In: *TPAMI*. 10.1109/TPAMI.2010.54 21088318

[CR20] Budvytis, I., Teichmann, M., Vojir, T., & Cipolla, R. (2019). Large scale joint semantic re-localisation and scene understanding via globally unique instance coordinate regression. In *BMVC*.

[CR21] Camposeco, F., Cohen, A., Pollefeys, M., & Sattler, T. (2019). Hybrid scene compression for visual localization. In *The IEEE conference on computer vision and pattern recognition (CVPR)*.

[CR22] Cao, S.,& Snavely, N. (2013). Graph-based discriminative learning for location recognition. In *CVPR*.

[CR23] Cao, S.,& Snavely, N. (2014). Minimal scene descriptions from structure from motion models. In *CVPR*.

[CR24] Carlevaris-Bianco N, Ushani AK, Eustice RM (2016). University of Michigan North Campus long-term vision and lidar dataset. IJRR.

[CR25] Castle, R. O., Klein, G., & Murray, D. W. (2008). Video-rate localization in multiple maps for wearable augmented reality. In *ISWC*.

[CR26] Cavallari, T., Bertinetto, L., Mukhoti, J., Torr, P.,& Golodetz, S. (2019). Let’s take this online: Adapting scene coordinate regression network predictions for online RGB-D camera relocalisation. In *3DV*.

[CR27] Cavallari, T., Golodetz, S., Lord, N. A., Valentin, J., Di Stefano, L., & Torr, P. H. S. (2017). On-the-fly adaptation of regression forests for online camera relocalisation. In *CVPR*.

[CR28] Cavallari, T., Golodetz, S., Lord, N., Valentin, J., Prisacariu, V., Di Stefano, L., & Torr, P. H. S. (2019). Real-time RGB-D camera pose estimation in novel scenes using a relocalisation cascade. In *TPAMI*. 10.1109/TPAMI.2019.2915068 31059430

[CR29] Chen, Z., Jacobson, A., Sünderhauf, N., Upcroft, B., Liu, L., Shen, C., Reid, I. D., & Milford., M. (2017). Deep learning features at scale for visual place recognition. In *ICRA*.

[CR30] Chen, D. M., Baatz, G., Köser, K., Tsai, S. S., Vedantham, R., Pylvänäinen, T., Roimela, K., Chen, X., Bach, J., Pollefeys, M., Girod, B.,& Grzeszczuk, R. (2011). City-scale landmark identification on mobile devices. In *CVPR*.

[CR31] Cheng, W., Lin, W., Chen, K., & Zhang, X. (2019). Cascaded parallel filtering for memory-efficient image-based localization. In *IEEE international conference on computer vision (ICCV)*.

[CR32] Choudhary, S., & Narayanan, P. J. (2012). Visibility probability structure from SFM datasets and applications. In *ECCV*.

[CR33] Chum O, Matas J (2008). Optimal randomized RANSAC. PAMI.

[CR34] Clark, R., Wang, S., Markham, A., Trigoni, N., & Wen, H. (2017). VidLoc: A deep spatio-temporal model for 6-DoF video-clip relocalization. In *CVPR*.

[CR35] Crandall, D., Owens, A., Snavely, N., & Huttenlocher, D. P. (2011). Discrete-continuous optimization for large-scale structure from motion. In *CVPR*. 10.1109/TPAMI.2012.218 24136425

[CR36] Dai, A., Nießner, M., Zollöfer, M., Izadi, S., & Theobalt, C. (2017). Bundle fusion: Real-time globally consistent 3D reconstruction using on-the-fly surface re-integration. In *ACM transactions on graphics 2017 (TOG)*.

[CR37] Davison AJ, Reid ID, Molton ND, Stasse O (2007). MonoSLAM: Real-time single camera SLAM. IEEE Transactions on Pattern Analysis and Machine Intelligence.

[CR38] Deng, J., Dong, W., Socher, R., Li, L.-J., Li, K.,& Fei-Fei, L. (2009). ImageNet: A large-scale hierarchical image database. In *CVPR*.

[CR39] DeTone, D., Malisiewicz, T., & Rabinovich, A. (2018). SuperPoint: Self-supervised interest point detection and description. In *The IEEE conference on computer vision and pattern recognition (CVPR) workshops*.

[CR40] Ding, M., Wang, Z., Sun, J., & Shi, J. & Luo, P. (2019). CamNet: Coarse-to-fine retrieval for camera re-localization. In *ICCV*.

[CR41] Donoser, M., & Schmalstieg, D. (2014). Discriminative feature-to-point matching in image-based locallization. In *CVPR*.

[CR42] Dusmanu, M., Rocco, I., Pajdla, T., Pollefeys, M., Sivic, J., Torii, A., & Sattler, T. (2019). D2-Net: A trainable CNN for joint detection and description of local features. In *CVPR*.

[CR43] Dusmanu, M., Rocco, I., Pajdla, T., Pollefeys, M., Sivic, J., Torii, A., & Sattler, T. (2019, June). D2-net: A trainable CNN for joint description and detection of local features. In *The IEEE conference on computer vision and pattern recognition (CVPR)*.

[CR44] DuToit, R. C., Hesch, J. A., Nerurkar, E. D., & Roumeliotis, S. I. (2017). Consistent map-based 3D localization on mobile devices. In *2017 IEEE international conference on robotics and automation (ICRA)*.

[CR45] Dymczyk, M., Lynen, S., Cieslewski, T., Bosse, M., Siegwart, R., & Furgale, P. (2015). The gist of maps—Summarizing experience for lifelong localization. In *2015 IEEE international conference on robotics and automation (ICRA)*.

[CR46] Ebel, P., Mishchuk, A., Yi, K. M., Fua, P., & Trulls, E. (2019). Beyond cartesian representations for local descriptors. In *The IEEE international conference on computer vision (ICCV)*.

[CR47] Fischler MA, Bolles RC (1981). Random sample consensus: A paradigm for model fitting with applications to image analysis and automated cartography. Communications of the ACM.

[CR48] Garg S, Suenderhauf N, Milford M (2019). Semantic-geometric visual place recognition: A new perspective for reconciling opposing views. The International Journal of Robotics Research.

[CR49] Germain, H., Bourmaud, G., & Lepetit, V. (2019). Sparse-to-dense hypercolumn matching for long-term visual localization. In *International conference on 3D vision (3DV)*.

[CR50] Haralick RM, Lee C-N, Ottenberg K, Nölle M (1994). Review and analysis of solutions of the three point perspective pose estimation problem. IJCV.

[CR51] Hartley R, Zisserman A (2003). Multiple view geometry in computer vision.

[CR52] Heng, L., Choi, B., Cui, Z., Geppert, M., Hu, S., Kuan, B., et al. (2019). Project AutoVision: Localization and 3D scene perception for an autonomous vehicle with a multi-camera system. In *2019 IEEE international conference on robotics and automation (ICRA)*.

[CR53] Hinterstoisser, S., Lepetit, V., Ilic, S., Holzer, S., Bradski, G., Konolige, K., & Navab, N. (2012). Model based training, detection and pose estimation of texture-less 3D objects in heavily cluttered scenes. In K. M. Lee, Y. Matsushita, J. M. Rehg, & Z. Hu (Eds.) *ACCV*.

[CR54] Huang, Z., Xu, Y., Shi, J., Zhou, X., Bao, H., & Zhang, G. (2019). Prior guided dropout for robust visual localization in dynamic environments. In *IEEE international conference on computer vision* (ICCV).

[CR55] Irschara, A., Zach, C., Frahm, J.-M.,& Bischof, H. (2009). From structure-from-motion point clouds to fast location recognition. In *CVPR*.

[CR56] Jones ES, Soatto S (2011). Visual-inertial navigation, mapping and localization: A scalable real-time causal approach. International Journal of Robotics Research.

[CR57] Kasyanov, A., Engelmann, F., Stückler, J.,& Leibe, B. (2017). Keyframe-based visual-inertial online slam with relocalization. In *2017 IEEE/RSJ international conference on intelligent robots and systems (IROS)*.

[CR58] Kazhdan M, Hoppe H (2013). Screened Poisson surface reconstruction. ACM Transactions on Graphics.

[CR59] Kendall, Alex, & Cipolla, Roberto. (2017). Geometric loss functions for camera pose regression with deep learning. In *CVPR*.

[CR60] Kendall, A., Grimes, M., & Cipolla, R. (2015). Posenet: A convolutional network for real-time 6-DoF camera relocalization. In *International conference on computer vision (ICCV)* (pp. 2938–2946).

[CR61] Kneip, L., Scaramuzza, D., & Siegwart, R. (2011). A novel parametrization of the perspective-three-point problem for a direct computation of absolute camera position and orientation. In *IEEE conference on computer vision and pattern recognition (CVPR)* (pp. 2969–2976).

[CR62] Kukelova, Z., Bujnak, M., & Pajdla, T. (2010). Closed-form solutions to minimal absolute pose problems with known vertical direction. In *ACCV*.

[CR63] Kukelova, Z., Bujnak, M.,& Pajdla, T. (2013). Real-time solution to the absolute pose problem with unknown radial distortion and focal length. In *ICCV*.

[CR64] Larsson, C. T. V., Fredriksson, J., & Kahl, F. (2016). Outlier rejection for absolute pose estimation with known orientation. In *BMVC*.

[CR65] Larsson, V., Kukelova, Z., & Zheng, Y. (2017). Making minimal solvers for absolute pose estimation compact and robust. In *ICCV*.

[CR66] Larsson, M. Stenborg, E., Toft, C., Hammarstrand, L., Sattler, T., & Kahl, F. (2019). Fine-grained segmentation networks: Self-supervised segmentation for improved long-term visual localization. In *IEEE international conference on computer vision (ICCV)*.

[CR67] Laskar, Z., Melekhov, I., Kalia, S., & Kannala, J. (2017). Camera relocalization by computing pairwise relative poses using convolutional neural network. In *ICCV workshops*.

[CR68] Lebeda, K., Matas, J. E. S., & Chum, O. (2012). Fixing the locally optimized RANSAC. In *British machine vision conference (BMVC)*.

[CR69] Li, Y., Snavely, N., Huttenlocher, D., & Fua, P. (2012). Worldwide pose estimation using 3D point clouds. In *ECCV*.

[CR70] Li, Y., Snavely, N., & Huttenlocher, D. P. (2010). Location recognition using prioritized feature matching. In *ECCV*.

[CR71] Lim, H., Sinha, S. N., Cohen, M. F., & Uyttendaele, M. (2012). Real-time image-based 6-DOF localization in large-scale environments. In *CVPR*.

[CR72] Liu, L., Li, H., & Dai, Y. (2017). Efficient global 2D–3D matching for camera localization in a large-scale 3D map. In *ICCV*.

[CR73] Lowe DG (2004). Distinctive image features from scale-invariant keypoints. The International Journal of Computer Vision.

[CR74] Lynen, S., Sattler, T., Bosse, M., Hesch, J., Pollefeys, M., & Siegwart, R. (2015). Get out of my lab: Large-scale real-time visual-inertial localization. In *Robotics: Science and systems (RSS)*.

[CR75] Maddern W, Pascoe G, Linegar C, Newman P (2017). 1 year, 1000 km: The Oxford RobotCar dataset. The International Journal of Robotics Research.

[CR76] Massiceti, D., Krull, A., Brachmann, E., Rother, C., & Torr, P. H. S. (2017). Random forests versus neural networks—What’s best for camera relocalization? In *ICRA*.

[CR77] Melekhov, I., Ylioinas, J., Kannala, J., & Rahtu, E. (2017). Image-based Localization using Hourglass Networks. In *ICCV workshops*.

[CR78] Meng, L., Chen, J., Tung, F., Little, J. J., Valentin, J., & de Silva, C. W. (2017). Backtracking regression forests for accurate camera relocalization. In *IROS*.

[CR79] Meng, L., Tung, F., Little, J. J., Valentin, J., & de Silva, C. W. (2018). Exploiting points and lines in regression forests for RGB-D camera relocalization. In *IROS*.

[CR80] Middelberg, S., Sattler, T., Untzelmann, O., & Kobbelt, L. (2014). Scalable 6-DOF localization on mobile devices. In *ECCV*.

[CR81] Milford, M. J, & Wyeth, G. F. (2012). SeqSLAM: Visual route-based navigation for sunny summer days and stormy winter nights. In *ICRA*.

[CR82] Mishchuk, A., Mishkin, D., Radenovic, F., & Matas, J. (2017). Working hard to know your neighbor’s margins: Local descriptor learning loss. In *Advances in neural information processing systems*.

[CR83] Mur-Artal R, Tardós JD (2017). ORB-SLAM2: An open-source SLAM system for monocular, stereo, and RGB-D cameras. IEEE Transactions on Robotics.

[CR84] Mur-Artal R, Tardós JD (2017). Visual-inertial monocular SLAM with map reuse. IEEE Robotics and Automation Letters.

[CR85] Naseer, T., Oliveira, G. L., Brox, T., & Burgard, W. (2017). Semantics-aware visual localization under challenging perceptual conditions. In *ICRA*.

[CR86] Newcombe, R. A., Izadi, S., Hilliges, O., Kim, D., Davison, A. J., & Kohli, P., Fitzgibbon, A. (2011). KinectFusion: Real-time dense surface mapping and tracking. In *IEEE ISMAR*.

[CR87] Noh, H., Araujo, A., Sim, J., Weyand, T., & Han, B. (2017). Large-scale image retrieval with attentive deep local features. In *International conference on computer vision (ICCV)* (pp. 3476–3485).

[CR88] Ono, Y., Trulls, E., Fua, P., & Yi, K. M. (2018). LF-Net: Learning local features from images. In *Advances in neural information processing systems* (Vol. 31).

[CR89] Pittaluga, F., Koppal, S. J., Kang, S. B., & Sinha, S. N. (2019, June). Revealing scenes by inverting structure from motion reconstructions. In *IEEE conference on computer vision and pattern recognition (CVPR)*.

[CR90] Radenović F, Tolias G, Chum O (2019). Fine-tuning CNN image retrieval with no human annotation. IEEE Transactions on Pattern Analysis and Machine Intelligence.

[CR91] Radwan N, Valada A, Burgard W (2018). VLocNet++: Deep multitask learning for semantic visual localization and odometry. RA-L.

[CR92] Raguram R, Chum O, Pollefeys M, Matas J, Frahm J-M (2013). USAC: A universal framework for random sample consensus. TPAMI.

[CR93] Revaud, J., Weinzaepfel, P., de Souza, C. R., & Humenberger, M. (2019). R2D2: Repeatable and reliable detector and descriptor. In *NeurIPS*.

[CR94] Robertson, D.,& Cipolla, R. (2004). An image-based system for urban navigation. In *BMVC*.

[CR95] Ronneberger, O., Fischer, P., & Brox, T. (2015). U-net: Convolutional networks for biomedical image segmentation. In *International conference on medical image computing and computer-assisted intervention*.

[CR96] Rublee, Ethan, Rabaud, Vincent, Konolige, Kurt, & Bradski, Gary. (2011). ORB: An efficient alternative to SIFT or SURF. In *The International Conference on Computer Vision (ICCV)* (pp. 2564–2571).

[CR97] Saha, S., & Varma, G., Jawahar, C. V. (2018). Improved visual relocalization by discovering anchor points. In *BMVC*.

[CR98] Sarlin, P.-E., Cadena, C., Siegwart, R., & Dymczyk, M. (2019). From coarse to fine: Robust hierarchical localization at large scale. In *The IEEE conference on computer vision and pattern recognition (CVPR)*.

[CR99] Sarlin, P.-E., DeTone, D., Malisiewicz, T., & Rabinovich, A. (2020). SuperGlue: Learning feature matching with graph neural networks. In *The IEEE conference on computer vision and pattern recognition (CVPR)*.

[CR100] Sattler, T. (2019). RansacLib—A template-based SAC implementation. https://github.com/tsattler/RansacLib.

[CR101] Sattler, T., Leibe, B., & Kobbelt, L. (2011). Fast image-based localization using direct 2D-to-3D matching. In *ICCV*.

[CR102] Sattler, T., Maddern, W., Toft, C., Torii, A., Hammarstrand, L., Stenborg, E., Safari, D., Okutomi, M., Pollefeys, M., Sivic, J., Kahl, F., & Pajdla, T.S. (2018). Benchmarking 6DOF outdoor visual localization in changing conditions. In *IEEE conference on computer vision and pattern recognition (CVPR)* (pp. 8601–8610).

[CR103] Sattler, T., Torii, A., Sivic, J., Pollefeys, M., Taira, H., Okutomi, M., & Pajdla, T. (2017). Are large-scale 3D models really necessary for accurate visual localization? In *CVPR*. 10.1109/TPAMI.2019.2941876 31535984

[CR104] Sattler, T., Weyand, T., Leibe, B., & Kobbelt, L. (2012). Image retrieval for image-based localization revisited. In *British machine vision conference (BMVC)*.

[CR105] Sattler, T., Zhou, Q., Pollefeys, M., & Leal-Taixe, L. (2019). Understanding the limitations of CNN-based absolute camera pose regression. In *CVPR*.

[CR106] Sattler T, Leibe B, Kobbelt L (2017). Efficient & effective prioritized matching for large-scale image-based localization. PAMI.

[CR107] Schneider T, Dymczyk M, Fehr M, Egger K, Lynen S, Gilitschenski I (2018). Maplab: An open framework for research in visual-inertial mapping and localization. IEEE Robotics and Automation Letters.

[CR108] Schönberger, J. L., & Frahm, J.-M. (2016). Structure-from-motion revisited. In *IEEE conference on computer vision and pattern recognition (CVPR)*.

[CR109] Schönberger, J. L., Zheng, E., Pollefeys, M., & Frahm, J.-M. (2016). Pixelwise view selection for unstructured multi-view stereo. In *European conference on computer vision (ECCV)*.

[CR110] Schönberger, J. L., Pollefeys, M., Geiger, A.,& Sattler, T. (2018). Semantic visual localization. In *CVPR*.

[CR111] Schops, T., Sattler, T., & Pollefeys, M. (2019). BAD SLAM: bundle adjusted direct RGB-D SLAM. In *The IEEE conference on computer vision and pattern recognition (CVPR)*.

[CR112] Se, S., Lowe, D., & Little, J. (2002). Global localization using distinctive visual features. In *IEEE/RSJ international conference on intelligent robots and systems*.

[CR113] Seymour, Z., Sikka, K., Chiu, H.-P., Samarasekera, S., & Kumar, R. (2019). Semantically-aware attentive neural embeddings for image-based visual localization. In *BMVC*.

[CR114] Shan, Q., Wu, C., Curless, B., Furukawa, Y., Hernandez, C., & Seitz, S. M. (2014). Accurate geo-registration by ground-to-aerial image matching. In *3DV*.

[CR115] Shi, T., Shen, S., Gao, X.,& Zhu, L. (2019). Visual localization using sparse semantic 3D map. In *2019 IEEE international conference on image processing (ICIP)*.

[CR116] Shotton, J., Glocker, B., Zach, C., Izadi, S., Criminisi, A., & Fitzgibbon, A. (2013). Scene coordinate regression forests for camera relocalization in RGB-d images. In *IEEE conference on computer visual pattern recogntion (CVPR)*.

[CR117] Sibbing, D., Sattler, T., Leibe, B.,& Kobbelt, L. (2013). SIFT-realistic rendering. In *3DV*.

[CR118] Simonyan K, Vedaldi A, Zisserman A (2014). Learning local feature descriptors using convex optimisation. TPAMI.

[CR119] Simo-Serra, E., Trulls, E., Ferraz, L., Kokkinos, I., Fua, P., & Moreno-Noguer, F. (2015). Discriminative learning of deep convolutional feature point descriptors. In *ICCV*.

[CR120] Snavely N, Seitz SM, Szeliski R (2008). Modeling the world from internet photo collections. IJCV.

[CR121] Stenborg, E., Toft, C.,& Hammarstrand, L. (2018). Long-term visual localization using semantically segmented images. In *2018 IEEE international conference on robotics and automation (ICRA)*.

[CR122] Sun, X., Xie, Y., Luo, P., & Wang, L. (2017). A dataset for benchmarking image-based localization. In *CVPR*.

[CR123] Sünderhauf, N., Shirazi, S., Jacobson, A., Dayoub, F., Pepperell, E., Upcroft, B., et al. (2015). Place recognition with ConvNet landmarks: Viewpoint-robust, condition-robust, training-free. In *Robotics: science and systems (RSS)*.

[CR124] Svärm L, Enqvist O, Kahl F, Oskarsson M (2017). City-scale localization for cameras with known vertical direction. PAMI.

[CR125] Taira, H., Okutomi, M., Sattler, T., Cimpoi, M., Pollefeys, M., Sivic, J., Pajdla, T., & Torii, A. (2018). Inloc: Indoor visual localization with dense matching and view synthesis. In *IEEE conference on computer vision and pattern recognition (CVPR)*. 10.1109/TPAMI.2019.2952114 31722474

[CR126] Taira, H., Rocco, I., Sedlar, J., Okutomi, M., Sivic, J., Pajdla, T., Sattler, T., & Torii, A. (2019). Is this the right place? Geometric-semantic pose verification for indoor visual localization. In *International conference on computer vision (ICCV)*.

[CR127] Tian, Y., Fan, B., & Wu, F. (2017). L2-Net: Deep learning of discriminative patch descriptor in Euclidean space. In *The IEEE conference on computer vision and pattern recognition (CVPR)*.

[CR128] Tian, Y., Yu, X., Fan, B., Wu, F., Heijnen, H., & Balntas, V. (2019). SOSNet: Second order similarity regularization for local descriptor learning. In *The IEEE conference on computer vision and pattern recognition (CVPR)*.

[CR129] Toft, C., Olsson, C., & Kahl, F. (2017). Long-term 3D localization and pose from semantic labellings. In *ICCV Workshops*.

[CR130] Toft, C., Stenborg, E., Hammarstrand, L., Brynte, L., Pollefeys, M., Sattler, T., & Kahl, F. (2018). Semantic match consistency for long-term visual localization. In *The European conference on computer vision (ECCV)*.

[CR131] Torii, A., Sivic, J.,& Pajdla, T. (2011). Visual localization by linear combination of image descriptors. In *Proceedings of the 2nd IEEE workshop on mobile vision, with ICCV*.

[CR132] Torii A, Arandjelovic R, Sivic J, Okutomi M, Pajdla T (2018). 24/7 place recognition by view synthesis. IEEE Transactions on Pattern Analysis and Machine Intelligence.

[CR133] Torii A, Sivic J, Okutomi M, Pajdla T (2015). Visual place recognition with repetitive structures. IEEE Transactions on Pattern Analysis and Machine Intelligence.

[CR134] Valada, A., Radwan, N., & Burgard, W. (2018). Deep auxiliary learning for visual localization and odometry. In *ICRA*.

[CR135] Valentin, J., Dai, A., Niessner, M., Kohli, P., Torr, P., Izadi, S., & Keskin, C. (2016). Learning to navigate the energy landscape. In *3D Vision (3DV)* (pp. 323–332).

[CR136] Valentin, J., Nießner, M., Shotton, J., Fitzgibbon, A., Izadi, S., & Torr, P. (2015). Exploiting uncertainty in regression forests for accurate camera relocalization. In *CVPR*.

[CR137] Valentin, J., Dai, A., Niessner, M., Kohli, P., Torr, P., Izadi, S.,& Keskin, C. (2016). Learning to navigate the energy landscape. In *International conference on 3D vision (3DV)*.

[CR138] Ventura J, Arth C, Reitmayr G, Schmalstieg D (2014). Global localization from monocular SLAM on a mobile phone. IEEE Transactions on Visualization and Computer Graphics.

[CR139] Walch, F., Hazirbas, C., Leal-Taixé, L., Sattler, T., Hilsenbeck, S., & Cremers, D. (2017). Image-based localization using LSTMs for structured feature correlation. In *ICCV*.

[CR140] Wang, Q., Zhou, X., Hariharan, B., & Snavely, N. (2020). Learning feature descriptors using camera pose supervision. arXiv:2004.13324.

[CR141] Wang, P., Huang, X., Cheng, X., Zhou, D., Geng, Q., & Yang, R. (2019). The ApolloScape open dataset for autonomous driving and its application. *IEEE Transactions on Pattern Analysis and Machine Intelligence*. 10.1109/TPAMI.2019.2926463. 10.1109/TPAMI.2019.2926463 31283496

[CR142] Williams, B., Klein, G.,& Reid, I. (2007). Real-time SLAM relocalisation. In *ICCV*. 10.1109/TPAMI.2011.41 21358004

[CR143] Xue, F., Wang, X., Yan, Z., Wang, Q., Wang, J., & Zha, H. (2019). Local supports global: Deep camera relocalization with sequence enhancement. In *IEEE international conference on computer vision (ICCV)*.

[CR144] Yang, L., Bai, Z., Tang, C., Li, H., Furukawa, Y., & Tan P. (2019). SANet: Scene agnostic network for camera localization. In *ICCV*.

[CR145] Yang, T.-Y., Nguyen, D.-K., Heijnen, H., & Balntas, V. (2020). UR2KiD: unifying retrieval, keypoint detection, and keypoint description without local correspondence supervision. arXiv:2001.07252.

[CR146] Yu, X., Chaturvedi, S., Feng, C., Taguchi, Y., Lee, T., Fernandes, C., & Ramalingam, S. (2018). VLASE: Vehicle localization by aggregating semantic edges. In *2018 IEEE/RSJ International Conference on Intelligent Robots and Systems (IROS)*.

[CR147] Zeisl, B., Sattler, T., & Pollefeys, M. (2015). Camera pose voting for large-scale image-based localization. In *ICCV*.

[CR148] Zhang, W.,& Kosecka, J. (2006). Image based localization in urban environments. In *International symposium on 3D data processing, visualization, and transmission (3DPVT)*.

[CR149] Zhang, J., Sun, D., Luo, Z., Yao, A., Zhou, L., Shen, T., et al. (2019). Learning two-view correspondences and geometry using order-aware network. In *IEEE international conference on computer vision (ICCV)*. 10.1109/TPAMI.2020.3048013 33373296

[CR150] Zheng, E., & Wu, C. (2015). Structure from motion using structure-less resection. In *The IEEE international conference on computer vision (ICCV). *

[CR151] Zhou, L., Luo, Z., Shen, T., Zhang, J., Zhen, M., Yao, Y., Fang, T., & Quan, L. (2020). KFNet: Learning temporal camera relocalization using Kalman Filtering. In *The IEEE conference on computer vision and pattern recognition (CVPR)*.

[CR152] Zhou, H., Sattler, T., & Jacobs, D. W. (2016). Evaluating local features for day-night matching. In *Proceedings of the ECCV workshops*.

[CR153] Zhou, Q., Sattler, T., Pollefeys, M., & Leal-Taixe, L. (2019). Visual localization from essential matrices: To learn or not to learn. In *IEEE international conference on robotics and automation (ICRA)*.

